# Fast quantitative time lapse displacement imaging of endothelial cell invasion

**DOI:** 10.1371/journal.pone.0227286

**Published:** 2020-01-07

**Authors:** Christian Steuwe, Marie-Mo Vaeyens, Alvaro Jorge-Peñas, Célie Cokelaere, Johan Hofkens, Maarten B. J. Roeffaers, Hans Van Oosterwyck

**Affiliations:** 1 Centre for Membrane Separations, Adsorption, Catalysis and Spectroscopy for Sustainable Solutions (cMACS), Department of Microbial and Molecular Systems (M^2^S), KU Leuven, Leuven, Belgium; 2 Biomechanics Section (BMe), Department of Mechanical Engineering, KU Leuven, Leuven, Belgium; 3 Molecular Imaging and Photonics, Department of Chemistry, KU Leuven, Leuven, Belgium; 4 Prometheus, Division of Skeletal Tissue Engineering, KU Leuven, Leuven, Belgium; Pennsylvania State Hershey College of Medicine, UNITED STATES

## Abstract

In order to unravel rapid mechano-chemical feedback mechanisms in sprouting angiogenesis, we combine selective plane illumination microscopy (SPIM) and tailored image registration algorithms — further referred to as SPIM-based displacement microscopy — with an *in vitro* model of angiogenesis. SPIM successfully tackles the problem of imaging large volumes while upholding the spatial resolution required for the analysis of matrix displacements at a subcellular level. Applied to *in vitro* angiogenic sprouts, this unique methodological combination relates subcellular activity — minute to second time scale growing and retracting of protrusions — of a multicellular systems to the surrounding matrix deformations with an exceptional temporal resolution of 1 minute for a stack with multiple sprouts simultaneously or every 4 seconds for a single sprout, which is 20 times faster than with a conventional confocal setup. Our study reveals collective but non-synchronised, non-continuous activity of adjacent sprouting cells along with correlations between matrix deformations and protrusion dynamics.

## Introduction

Traction Force Microscopy (TFM) is a powerful tool to study cell-matrix mechanical interactions, as it allows to quantify unknown cellular forces (tractions) from the displacements (deformations) they induce in the extracellular matrix, provided the mechanical (elastic) properties of the matrix are known. While the technique is well established for 2D cell culture systems (i.e. for cells cultured on relatively soft, elastic substrates) [1–4], resolving tractions for 3D cultures (with cells being embedded in a matrix-mimicking hydrogel) is computationally much more challenging. It requires the use of numerical methods (such as the finite element method) to solve the inverse mechanical problem that underlies TFM and that relates unknown tractions to measured displacements [5–7]. The fact that the measured displacements contain errors (e.g. due to a limited number of sampling points and due to microscopy limitations), renders the inverse problem ill-posed, thereby requiring proper regularization to avoid error propagation from displacements to tractions [8]. One can avoid the computationally challenging step of 3D traction recovery (or 4D, when also resolving in time) by only solving for matrix deformations (by means of image registration) and expressing cell-matrix mechanical interactions in terms of matrix deformation-based metrics [9]. This so called displacement microscopy–combining live cell optical microscopy with image registration—has the additional advantage that mechanical characterisation of the matrix is not required (provided all experiments are performed in one and the same matrix).

Traction force and displacement microscopy techniques typically rely on point scanning techniques. Much faster imaging can be provided with Selective Plane Illumination Microscopy (SPIM). SPIM [10–12] uses a sheet of light to illuminate samples, as proposed by Voie *et al*. [13] and further improved by the Stelzer group [14,15]. While SPIM is generally regarded as a fast scanning technique compared to point scanning techniques [16,17], recent studies demonstrate even faster setups by using for example multiple sheets [18], and many extensions have been developed such as Limited-View LSM [19] and Tiling Lightsheet SPIM [20]. Fast and efficient illumination restricted to the observed focal volume additionally minimizes photobleaching and phototoxicity [21,22]. At the same time, live cell imaging with SPIM imposes design challenges [23–25] on the sample geometry that needs to be tailored to the SPIM setup. SPIM has been typically applied for imaging large biological samples such as embryos [14,23,26,27], large tissues such as mouse brain samples [28] and tissue mimics such as spheroids, organoids or cellular aggregates [24,25,29–31], with only a few studies reporting on single-cell SPIM [32–34].

So far SPIM has not been combined with any traction force or displacement microscopy, nor has it been used to study angiogenic sprout dynamics. In the context of angiogenesis—the growth of blood vessels from the pre-existing vasculature–only a few studies exist that have reported on displacements around angiogenic sprouts, again using point scanning techniques for live cell imaging [35,36]. However, fast interactions between matrix and sprout—with minute to second scale protrusions and retractions [37,38]—in larger multicellular volumes remain difficult to monitor with point scanning techniques because of the relatively slow scanning, but are vital for understanding sprout progression [39].

To fill this methodological gap, we developed a SPIM-compatible *in vitro* model of angiogenesis for applying SPIM-based displacement microscopy. While the signal leading to sprout initiation is a pro-angiogenic chemical signal—with Vascular Endothelial Growth Factor (VEGF) being the most prominent one [40]—it is well known that endothelial cells respond to mechanical cues (such as matrix stiffness) as well and need to apply forces to their environment in order to move forward [41–47]. The importance of these cell-matrix mechanical interactions for sprout progression explains the rationale for studying them by means of displacement microscopy. By applying SPIM-based displacement microscopy analysis to the *in vitro* model of angiogenesis, we demonstrate that sprouts do neither continuously nor synchronously exert forces on their surrounding extracellular matrix. Finally, we focus on short term dynamics of growing protrusions and validate that fast volumetric imaging is vital for following the sprout–matrix interaction during these processes at the minute to second time scale.

## Methods and materials

The study was approved by the Medical Ethics Commission of the KU Leuven University Hospitals (file number S54744).

**Fig 1:**
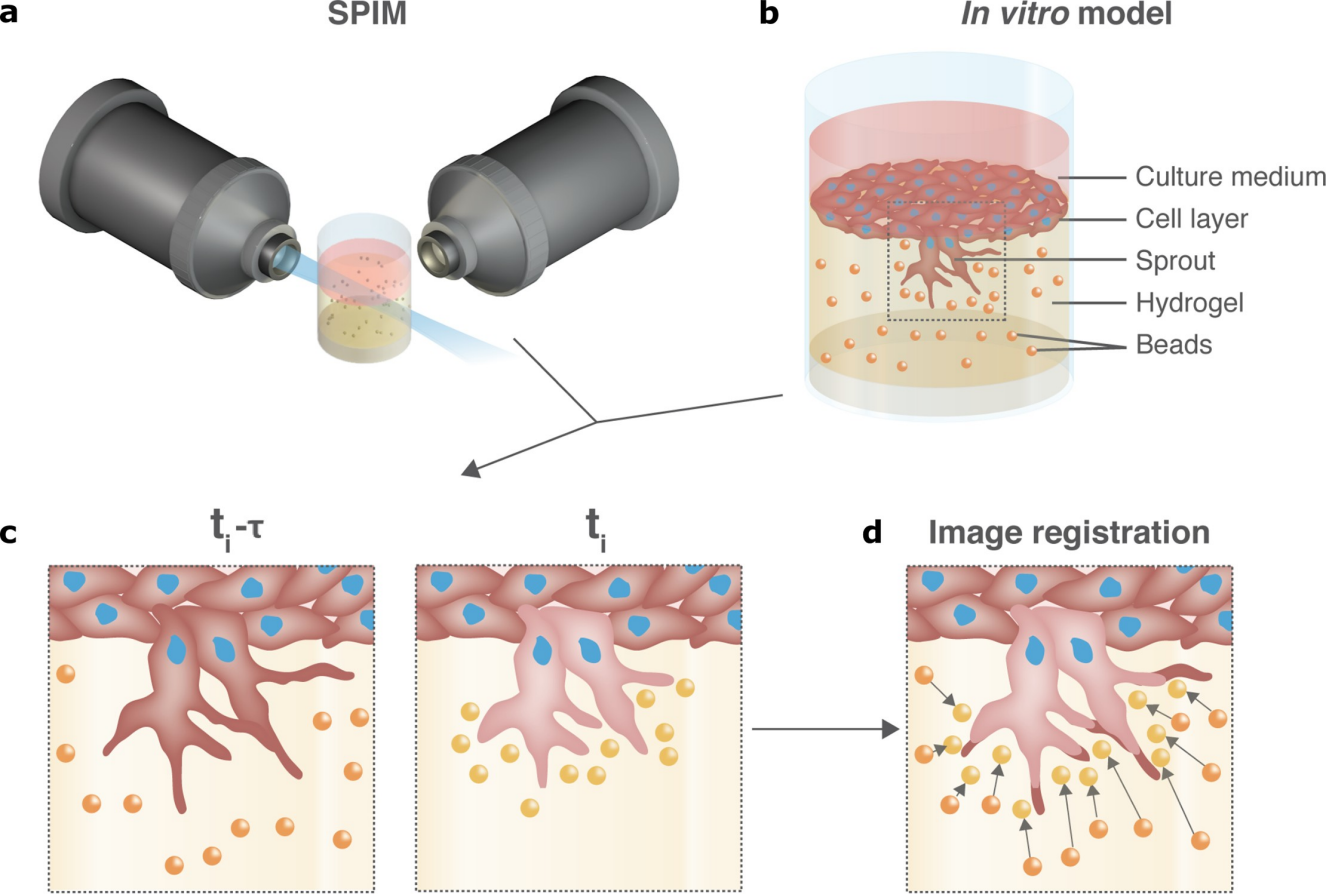
Workflow of 4D displacement microscopy around *in vitro* angiogenic sprouts. **(a)** SPIM setup with mounted sample (see S1 and S2 Figs for specifications). **(b)** SPIM-compatible i*n vitro* model of angiogenesis showing endothelial cells invading and sprouting into a collagen gel with embedded fluorescent beads. **(c)** Live fluorescence cell imaging of the sprouts and beads captures cell-matrix mechanical interactions and allows mapping sprout-induced matrix deformations by **(d)** registering the fluorescent beads and sprout segmentation in time.

First, to gain imaging speed and to increase the monitored volume, we replace point scanning imaging techniques [5,48] by fast plane scanning using orthogonal lenses as offered by SPIM (see Fig 1A and S1 and S2 Figs for the used SPIM setup). Next, we develop a robust, SPIM-compatible sample design for *in situ* 3D endothelial cells invading and forming sprouts into a collagen hydrogel with embedded fluorescent microspheres (beads) as fiducial markers (Fig 1B as well as S3 Fig). In this *in vitro* model sprouting is induced by sphingosine-1-phosphate (S1P), a pro-angiogenic factor that is added to the collagen matrix (as well as other pro-angiogenic growth factors that are present in the EGM2 culture medium). Sprouting tip cells then laterally inhibit neighbouring cells to become tip cells as well. The amount of sprouting (i.e. the amount of endothelial cells that turn from a quiescent to a migratory phenotype) is controlled by a number of signaling mechanisms that regulate lateral inhibition, one of the most studied ones being Dll4-Notch signaling [40,49,50]. Finally, we tailor displacement microscopy algorithms to calculate both absolute and incremental displacements (Fig 1C and 1D) of the collagen matrix. Due to its capability to deal with large distortions in complex materials[51], Free Form Deformation-based non-rigid image registration is used for both approaches to compare images of the fluorescent beads embedded in the collagen and extract the corresponding matrix displacement fields. Absolute displacements (t_ref_ = t_fix_) result from the registration and comparison of the image stack of fluorescent beads acquired under cell activity at each time point t_i_ against the (fixed reference) image stack acquired in a stress-free state of the hydrogel at timepoint t_fix_. The latter was obtained at the end of the experiment by adding Cytochalasin D [6,52] (CytoD), a cell-permeable mycotoxin, disrupting the cells’ actin cytoskeleton and relaxing the sample (see S4 Fig and S1 Video). At the same time, incremental displacements (t_ref_ = ti−τ) can be computed by registering image stacks of fluorescent beads acquired at sequential time points (non-fixed reference) spaced by a time interval *τ*. (See S5 Fig for notations and comparison of absolute versus incremental displacements). We highlight that both approaches are complementary: while absolute displacements are indicative for the direction and intensity with which cells are mechanically interacting with the matrix, the incremental displacements better reveal the temporal dynamics with which these interactions change. All methods are elaborated below in more detail.

### SPIM unit

The SPIM (or light sheet) imaging setup is based on a light sheet illuminating a thin (3 μm) section of the sample at the focal plane of the imaging objective (S1 and S2 Figs).

#### Light sheet generation of the SPIM microscope

In its simplest form, SPIM setups combine a single cylindrical lens together with an objective in a telescope configuration to generate a sheet/plane of light along the direction of the optical path. Here we decided to generate a virtual light sheet (S1 and S2 Figs) to yield a better-defined illuminated volume inside the sample. The virtual light sheet is formed by the beam waist of a focussed Gaussian beam that is being swept over a plane perpendicular to the imaging objective this way illuminating a planar volume inside the sample. If the sweeping frequency is synchronized and sufficiently high in comparison to the exposure time of the camera, the swept gaussian beam will appear as a plane of light. To deflect the beam angularly, we use a galvanometric mirror (62xxH series, Cambridge Technology, USA) with a suitable driver. A scan lens (f = 80 mm) converts the angular movement of the galvanometric mirror into a vertical, parallel translation. A tube lens (f = 200 mm) in combination with the illumination objective (Nikon Plan Flour 10x, NA = 0.3, working distance: 16 mm) acts as a telescope. Consequently, the objective back focal plane is effectively conjugated to the scanner. An angular beam tilt in the scanner plane therefore results in an angular beam tilt in the illumination objective back focal plane and is translated into a vertical translation behind the objective. The generated virtual Gaussian light sheet is adjusted to overlap with the focal plane of the imaging objective. In the vertical direction, this is done by adjusting an angular offset of the galvo mirror. In z-direction (perpendicular to the plane of the sheet), the adjustment is performed by changing the angle of a mirror in the conjugate plane of the illumination objective. The width of the Gaussian focus with NA = 0.3 is a nominal 820 nm with a length (confocal parameter) of just 4 μm. As light sheet imaging is a trade-off between field of view and light sheet thickness, we purposefully further reduced the numerical aperture with an iris to have a focus length of > 50 μm with a width of about 3 μm.

The waveform for driving the galvanometric mirror is customised and generated by a programme written in C#. The digital to analogue conversion is performed by a data acquisition card (NI USB-6341).

Two laser sources have been used throughout this study. For two-colour displacement imaging we employed a 488 nm Argon ion laser (Model 163-C1210, Spectra Physics, Mountain View, CA, USA). Note that the red fluorescent beads could also be sufficiently excited using this wavelength. For DAPI diffusion experiments we coupled a 375 nm UV diode laser into the system (Excelsior, 375 nm, Spectra Physics, Mountain View, CA, USA).

#### Detection subsystem

The imaging unit of the SPIM platform (S1 and S2 Figs) obeys the principles of a widefield fluorescence microscope. The sample is in first instance imaged by an objective lens (Nikon NIR APO, 40x, NA = 0.8, working distance: 3.5 mm). Our samples are completely immersed in water which demands an infinity corrected water dipping lens. Furthermore, for the live cell experiments we heat up the whole medium to 37 degrees. The contrast between cells and fiducial markers is generated by introducing two different fluophores, with green emission (LifeAct/GFP, 520 nm, F-actin of the cells) and red emission (605 nm, fluorescent beads). A tube lens (f = 200 mm) forms the primary image on two charge coupled device (CCD) chips of two separate cameras (Hamamatsu ImageEM CCD digital camera, resolution: 512×512 pixels, pixel pitch: 16 μm, frame rate: up to 33 frames per second (fps) in full frame mode, Hamamatsu Photonics K.K., Japan). Each of the cameras is synchronised to the sweeping frequency of the sheet generating signal to avoid random ghost images. A dichroic filter splits red and green emission light for dual colour imaging (600 nm, 25.2 x 35.6 mm, Techspec–Edmund optics). Residual excitation light is blocked by adequate filters for both emission channels. For the fluorescent proteins LifAct/GFP, we use a 500 nm long pass filter (Chroma, USA). For imaging the fluorescent fiducial marker beads, we use a 600 nm bandpass filter (FB600–40, Thorlabs). In order to overlay the two planes of both cameras, we used a sample containing beads only and removed the green bandpass filter (F1, Edmund #67–030, 520 nm FWHM:40 nm) and replaced it with a 500 nm long pass filter. The second camera was then overlaid with the first image by means of a micrometer stage, with an error limited to the optical resolution of the setup. The effective 20 nm broad spectral detection band for the fiducial markers provides strong signals and sufficient contrast. For DAPI we change the filter set of the green detection channel to a blue-shifted (440–20 nm) filter.

#### SPIM movement unit, sample containment and heating

We opted for sample movement, hence unlike on a confocal or wide field microscope, the sample is moved for acquiring three-dimensional image stacks. The sample is translated along the optical axis of the imaging unit (z-axis) and hence through the cross-section of the imaging focal and illumination plane. Since the z-movement is critical for reliable image acquisition we use a computer controlled piezo stage (Q-545.20, Physik Instrumente, Germany). Using this piezo and by carefully adapting its driver, we care for sample inertia. For X, Y and coarse Z movements, a compact 4 axis motor assembly USB-4D-Stage was used (Picard-Industries, USA). The objectives are mounted in a container of acrylic glass and sealed using vacuum grease. For live-cell experiments, cell medium is filled into the chamber and warmed up using a heating element. A proportional-integral-derivative (PID) controller was implemented on an Arduino Uno microcontroller board (S1 and S2 Figs). It gets feedback from a thermistor electronic element which is changing its resistance upon temperature variations. The Arduino output signal is amplified and controls the heating element. After a warming-up period and a short overshoot, the temperature regulation of the PID-controller is stable enough for live-cell experiments (S1 and S2 Figs).

#### SPIM acquisition and control software

We use Micro-Manager, a software package for the control and synchronisation of the SPIM platform parts. For this software package, a plugin was written by the developers of OpenSPIM. This plugin controls the cameras and sample stages (for xyz movement of the sample) to acquire three-dimensional stacks. The software was adapted for stable dual colour imaging. We also supplied an additional customised driver for the Physik Intrumente piezo stage. The waveform generation is not yet included in the Micro-Manager software but has its own graphical user interface. Similarly, the Arduino controller is read out simply by USB (RS232 protocol) and a terminal programme.

### *In vitro* angiogenesis model preparation

#### Cell culture and transduction

Pooled wild-type human vein umbilical cord (HUVEC) cells were purchased from Angio-Proteomie (Boston, USA). The cells were subcultured at approximately 90% confluency or used for displacement microscopy experiments up to passage 6. The cells were maintained in complete endothelial growth medium (EGM^™^-2 BulletKit^™^, Lonza, Switzerland), which was exchanged 3 times per week. Before live cell fluorescence microscopy, 70% confluent wild type HUVEC cells were transduced with adenoviral LifeAct-GFP2 (Ibidi, Martinsried, Germany) at multiplicity of infection (MOI) of 10 and incubated for 16 to 24 h. For diffusion experiments, non-fluorescent wild-type cells were stained with DAPI (Invitrogen, Belgium) *in situ*.

#### Rat tail collagen type I hydrogel preparation

Collagen mixes with a total volume of 1 ml before casting hydrogels in the tube containers were prepared on ice in a 50 ml conical tube by mixing collagen type I (Merck Millipore, Darmstadt, Germany) with 150 μl sodium bicarbonate buffer (15.6 mg/mL) and the appropriate variable amount of EGM2 containing (5 μl of) fluorescent bead solution (2% solids, 0.2 μm diameter, carboxylated, ex/em 580/605, Invitrogen, Belgium) to a final collagen concentration as indicated in the diffusion results, between 0.5 and 3 mg/ml. To initiate the polymerization reaction, a pH switch of the acidic collagen mixture was induced by using 1 M sodium hydroxide (Sodium hydroxide, VWR Chemicals, USA), after which the collagen was supplemented with pro-angiogenic factor Sphingosine-1-Phosphate (S1P, Sigma-Aldrich, Belgium) to a final concentration of 1 μM. Using a Pasteur pipette, the collagen was then introduced into the container to fill the 15 mm long part of the tube (S3 Fig). The hydrogels were allowed to polymerize and equilibrate in an incubator with 5% CO_2_ at 37°C for a minimum of 30 minutes, before proceeding with the cell seeding.

#### *In vitro* angiogenesis model tuned for SPIM

The *in vitro* angiogenesis model (S3 Fig) was an endothelial invasion assay where HUVEC cells, starting from a densely seeded cell layer, invade and form (~50–250 μm long) sprouts overnight within a 1.5 mg/ml collagen hydrogel containing pro-angiogenic factor S1P [53–57]. The physical sample setup was designed to meet the requirements for fast imaging with SPIM, which dictates that the sample has to be transparent from two sides. Cylindrical imaging chambers with 2 mm inner diameter were fabricated from laboratory grade tubing (Bola, S 1815–07). This fluoroplastic polymer (FEP) tube exhibits a refractive index which is similar to the one of water. Hence imaging aberrations are minimized when imaging through the plastic using a water dipping objective. The tube was sealed at the bottom employing two component glue and was sterilized by UV light (30 min) before hydrogel casting. An amount of about 10 μl of the collagen mixture was pipetted into the imaging tube. After polymerization as described previously, 60000 cells from a 70–90% confluent HUVEC culture in 10 μl of EGM2 were seeded through the opening and the tube was placed for 15 minutes in the incubator at 37°C, 5% CO_2_. This allowed the cells to sink and form a cellular layer on top of the collagen. Afterwards, abundant medium was added to the sample. The cells were then allowed to initiate invasion and form sprouts overnight before live imaging sprout dynamics.

### Imaging

#### Imaging single sprouts

Samples were transferred to the SPIM setup and live imaged at 37°C in M199 medium supplemented with 125 mM HEPES to buffer the medium to a neutral pH in ambient air. The fluorescence signals of the LifeAct-GFP2-cells and the beads were recorded using the setup described above. Spectral filtering ensured that beads and cells were imaged separately and simultaneously. Large volume timelapses consist of 200 x 200 x 200 μm^3^ volumes (512 pixels * 512 pixels * 201 planes), which were acquired for approximately 4h with temporal resolutions of 1 min (Res = 1 min). For local (thin volume) fast scanning, volumes of 200 x 200 x 20 μm^3^ (512 pixels * 512 pixels * 21 planes) with temporal resolutions up to 4 seconds (Res = 4 s) were recorded. In both scan modes, data was thus captured with voxel sizes of 0.39 x 0.39 x 1 μm^3^. To obtain the relaxed end state required to compute absolute displacements, cell relaxation was chemically induced by the actin cytoskeleton inhibitor cytochalasin D, and the gradual relaxation of cells was monitored during up to 4 hours of relaxation (S1 Video and S4 Fig).

#### Imaging DAPI diffusion experiments

To estimate the diffusion time preceding the chemically induced relaxing of the sprouts, experiments with 4’,6-diamidino-2-phenylindole (DAPI) were performed. DAPI is a cell-membrane permeable molecule (MW = 350 g/mol) that interacts with double stranded DNA which results in the formation of a fluorescent complex [58,59]. DAPI was added before imaging at final concentrations between 10 to 30 μM depending on the experiment. Subsequently, the samples were imaged for up to 3 hours. Fluorescence intensities were measured in ImageJ and plotted in Matlab (The MathWorks Inc., Natick, MA USA) fitting a diffusion model. The average inflection point was then estimated based on the distribution of inflection points (S4 Fig).

#### Imaging acellular control collagen samples

Cell-free control experiments were performed by recording timelapses of independent samples of representative collagen gels containing fluorescent beads but no cells. Two series of timings were chosen to mimic the cellular experiments: (1) 3D z-stacks of > 20 micron were acquired every 10–15 seconds for 2–3 hours. (2) Similar stacks were scanned every 4 seconds for 20 minutes. Other imaging parameters were similar to those from the cellular experiments.

### Computational analysis

#### Enhancement of bead images

The quality of the acquired z-stacks of the fluorescent beads has a great impact on the calculation of the cell-induced displacement fields. To boost blob-like structures and reduce noise, these images were processed by a difference of Gaussian filter and combined with a contrast stretching operation [51].

#### Cell mask calculation

To obtain a mask of the cellular structures, z—stacks containing sprouts were first enhanced by a two-step process consisting of a penalized least squares denoising [60] followed by a contrast stretching operation to smoothen and highlight the cell layer and sprouting cells from the background. Then, processed images were used to segment cellular structures by Otsu’s thresholding algorithm [61].

#### Sample drift compensation

Enhanced images of the fluorescent beads were used to compensate for the translational shifts in the experimental setup, including the drift of the microscope stage and/or global sample displacements caused by the activity of the cell layer present during the timelapse acquisitions. Phase-correlation-based rigid image registration was used to efficiently correct for the average global shift between images of fluorescent beads. Drift compensation is usually performed by rigidly registering (i.e. aligning) the bead images at every time point to the bead image of a pre-selected fixed time-point that acts as a global reference, which is commonly selected as the bead image after mechanical relaxation of cells. This strategy works fine for small hydrogel deformations showing a fast decay and thus, drift compensation algorithms benefit from having large regions with negligible bead displacements in the acquired field of view. However, this approach hinders the calculations under large deformations or when significant bead displacements are present within the whole field of view, as expected for images of multiple sprouts growing into a collagen gel. Instead, assuming that both experimental drifts and cell-induced hydrogel displacements will be smaller between consecutive time points than between each time point and the global reference, images of fluorescent beads on consecutive time points were rigidly registered to exploit the temporal coherency over long time lapse acquisitions. Finally, the position of rigidly registered images was expressed in a global frame of reference.

#### SPIM-based 4D displacement microscopy

Drift-compensated images of fluorescent beads were used to compute the local matrix displacements induced by the cell layer and the sprouts. The calculation of the 4D matrix displacements was formulated as a multiscale Free Form Deformation (FFD) non-rigid image registration process, which has been proven suitable for dealing with full-field deformations under large displacement and strain regimes[51]. Briefly, in FFD-based displacement field calculation, two bead images are compared, where one bead image is warped to match another fixed one (reference image). The algorithm overlays a regular grid over the reference image, of which the nodes are defined as the control points of multivariate B-splines curves. Then, the position of the control points is tuned iteratively during an optimization process, which warps the moving image until it matches the reference image. This yields as output a full displacement field, i.e. a vector value at each voxel of the registered images. Although the method warps the moving image to fit the reference image, both the underlying B-spline-based transformation model and the resulting displacement field are defined from the reference to moving image. To cope with different levels of matrix deformations while providing smooth displacement fields, the described FFD-based registration scheme was performed following a coarse-to-fine multiscale strategy. This implies that bead images were first registered using a relatively large grid size (i.e. few control points) and then, the output was used as the starting point for the next scale where the grid is refined, repeating the process iteratively for multiple refinement scales. Specifically, a three-level multiscale approach was used here with 180 x 180 x 44 voxels, 90 x 90 x 22 voxels, and 45 x 45 x 11 voxels grid size for the coarsest, intermediate and finest scales, respectively. During the optimization process that drives the registration by warping the moving image towards the reference image, a normalized correlation coefficient was used as the metric that iteratively calculates the similarity between both images. Furthermore, a stochastic gradient descent method with adaptive estimation of the step size was selected as optimizer due to its efficiency for registering very large image volumes: full-field displacement fields of 3D images containing ~512 x 512 x 200 voxels were obtained within 30 to 45 seconds in a standard Intel Core i7 PC.

#### Removal of engulfed beads from the calculation of the displacement fields

Beads engulfed by the cells move independently of the cell-matrix mechanical interactions. Thus, to remove these beads from further data analysis and minimize errors in the calculation of the matrix displacements, the binary mask of the segmented cellular structures (sprouts and cell layer) was supplied to the FFD-based displacement field calculation algorithm. This mask ensured that engulfed beads were not corrupting the matrix displacements in the surroundings to the cell-matrix interface.

#### Absolute versus incremental displacement fields

To obtain absolute displacement fields (S5B Fig), the images of fluorescent beads in the stressed hydrogel under the effects of cellular tractions were registered to the image of fluorescent beads in the relaxed hydrogel acquired after chemically inducing mechanical relaxation of the cells. Hence, the image of fluorescent beads at each time point showing a stressed hydrogel state acted as the moving image during the calculation of FFD-based displacements, and the one for the relaxed hydrogel as the (fixed) reference image (t_ref_ = t_fix_). Absolute displacements provide quantitative information on the magnitude, direction and global distribution of cell-matrix mechanical interactions and it is the standard approach in Traction Force Microscopy experiments. However, it might conceal the instantaneous cell-matrix interactions occurring between stressed hydrogel states along the timelapse. Therefore, to unravel the mechanical interactions among sprouts within a given time-lag *τ*, the incremental displacements were computed (S5B Fig), where the image of fluorescent beads at each time point t_i_ was used as the moving image and the one at time point ti−τ as the reference image in FFD-based registration (t_ref_ = ti−τ). In this way for every timepoint incremental displacement values are obtained compared to a time-lag *τ* earlier. Note that, while the magnitude of the incremental displacements reflects the instantaneous local changes in the displacement field within a time-lag *τ*, their direction should not be interpreted as the push-pull behavior of the sprouts (S5C and S5D Fig). Similar to absolute displacements, incremental displacements are computed at the same temporal resolution *Res* used during the acquisition, with *Res* *≤* *τ*. Therefore, the temporal dynamics of the cell-matrix mechanical interactions can be analyzed at different time scales (specified by *τ*) with a temporal resolution *Res* as good as the one provided by the imaging system.

#### Full-field and local rms displacements

To quantify the cell-matrix mechanical interaction over time, the root-mean-square (rms) displacement was computed from the displacement field for each time point in a selected region of interest as:

$$u_{\mathrm{rms}}=\sqrt{\frac{1}{N}\sum_{n=1}^{N}{\left\|U_{n}\right\|}_{2}^{2}}$$

with ‖***U**_n_*‖_2_ being the L_2_-norm of the displacement vector ***U***_*n*_ at location *n* within a region of interest defined by a total of *N* spatial points.

Two types of regions have been considered. A rectangular box around the sprout(s) of interest that provides the rms displacement of the full-field, and a mask covering the displacement field within a close vicinity (~ 6 micron) of the sprout surface, which provides a local rms displacement value. The local mask was obtained by a binary dilation operation applied to the cell mask of the selected sprout.

The full-field and local rms displacements have been computed for both absolute and incremental displacement fields.

#### Shape morphometry

The dynamics of the cell morphology were assessed qualitatively by contour evolution plots and quantitatively by the length of individual protrusions and the normalized volume difference over time.

Contour evolution plots display the changes of the cell shape over time by overlapping the color-coded contours of the Z-projected cell mask at different time points.

To calculate the length of a selected protrusion, the morphological skeleton of its Z-projected cell mask was first extracted. Then, the protrusion length at each time point was defined as the length of the shortest path within the corresponding skeleton from its base to its protrusion tip.

Finally, the normalized volume difference at each time point was obtained as:

$$V_{\mathrm{norm}\_\mathrm{dif}}=\frac{\sum \left(|M_{t_{i}}-M_{t_{\mathrm{ref}}}|>0\right)}{\sum \left(M_{t_{i}}>0\right)}$$

where $M_{t_{\mathrm{ref}}}$ and $M_{t_{i}}$ are the segmented cell masks at specified reference time point t_ref_ and at current time point t_i_, respectively. Analogously to the calculation of absolute and incremental displacement fields, the reference time point can be selected to be a fixed (t_ref_ = t_fix_) or relative (t_ref_ = ti—*τ*) time point.

#### Implementation and visualization

The computational workflow was implemented using custom-made code written in Matlab, except for FFD-based displacement field estimation, which was computed using the open-source software package elastix [62]. The integration of the elastix -based FFD image registration on the main computational workflow was done as previously described [51,63]. The software program Paraview 5.2 (Kitware Inc., NY USA) was used for the 3D rendering of segmented sprouts and the visualization of 3D vector fields.

### Validation

#### Stability of the experimental setup and noise level of the displacements

The SPIM-based displacement microscopy platform was extensively validated by control experiments with cell-free collagen samples. To confirm the stability of the experimental setup and its suitability for calculation of cell-induced matrix displacements, acellular control hydrogels with embedded fluorescent beads were prepared and imaged as previously described to check the existence of unwanted motion of beads. Acquired time-series of bead image volumes were first processed and compensated for possible stage drifts and then, analyzed by spatio-temporal image correlation spectroscopy (STICS) in 3D (S6 Fig). Briefly, the three-dimensional spatial correlation map between every bead image at a given time-lag Δ*t* apart from each other was computed and averaged in time. Then, the obtained averaged correlation peak was compared with the one obtained for Δ*t* = 0 (time averaged autocorrelation). Advecting beads would in general shift the averaged correlation peak over different values of Δt, while diffusing beads would broaden it. To quantify the peak location and width at each Δt, the mean and standard deviation of a fitted multidimensional Gaussian function was used. Finally, note that bead advection was *a priori* expected to be negligible for drift-compensated images of fluorescent beads.

Additionally, the stability of the bead locations was qualitatively assessed by building a kymographic representation, showing spatial bead distributions in time of randomly selected z-planes of the drift-compensated images of fluorescent beads (S7 Fig). If no bead motion occurred, the beads were stable over time, resulting in straight lines along the time axis of the kymograph. On the other hand, diffusing beads would show an oscillating line along the time axis, while advecting beads would show a drifted line along the time axis.

Finally, the local non-rigid displacements of the drift-compensated images of fluorescent beads were computed for the control experiments to detect the existence of spurious displacements and get the background noise level of the FFD-based image registration for the current experimental setup.

#### Correlations and sample sizes

Auto- and cross-correlations in S1 Table between the temporal evolution of rms displacements and morphological metrics were calculated in Matlab (The MathWorks Inc., Natick, MA USA). For assessing the diffusion rate of the system, measurements from five independent samples were considered. For the STICS analysis, based on the collagen control experiments, nine stacks gathered from three independent samples were analyzed, for each of the three collagen concentrations measured.

## Results and discussion

**Fig 2:**
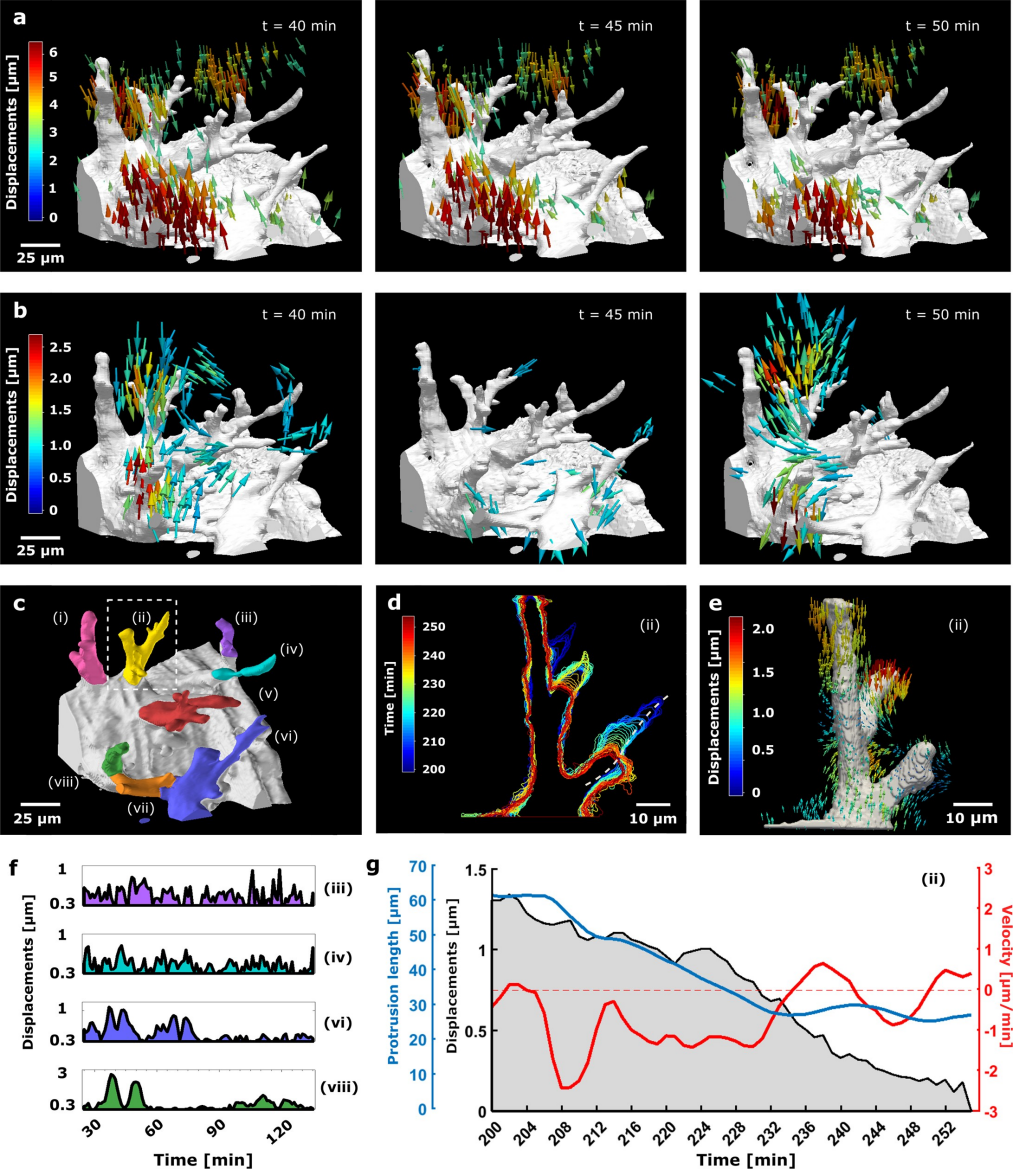
4D SPIM-based displacement microscopy enables matrix displacement quantification at high temporal resolution, simultaneously around multiple sprouts and in relation to protrusion dynamics. **(a)** Absolute (t_ref_ = t_fix_ at *t =* 255 min) and **(b)** incremental (t_ref_ = t_i−_τ,with τ = 5 min) displacements of indicated timepoints (see S5 Fig). For visualization purposes the smallest displacements (< 2 μm for (a) and < 0.75 μm for (b)) are not shown. For the full range of displacement magnitudes, see S6 Video. **(c)** Segmented and colour-coded sprouts. One selected sprout is marked by a dashed box. **(d)** Contour evolution plots of sprout (ii) marked in (c) and projected in z-direction and time, showing the dynamics of its protrusions during the final 55 minutes of the chemically-induced relaxation. (**e)** Absolute displacements near the surface of sprout c-(ii). **(f)** Incremental rms displacements as a function of time in close vicinity of 4 selected sprouts from (c) (see S8 Fig for additional graphs). **(g)** Activity profile during relaxation of sprout c-(ii) as assessed by its length (blue line; see also white dashed line in (d)) and velocity (red line) as a function of time. Protrusion velocity is positive for protrusion extension and negative for retraction. Absolute rms displacements in close vicinity of the selected protrusion are plotted as well (black shaded line). Absolute displacements are obtained from a stress-free state at t *=* 255 min after addition of 4 μM CytoD at t *=* 135 min. Acquired volume of ~200 x 200 x 200 micron^3^ with 1 minute temporal resolution.

While the assessment of overall sprout progression in the *in vitro* model of angiogenesis (that typically runs over 24 hours) does not require any high temporal resolutions (as sprout progression is typically expressed as growth/hour or growth/day), capturing the fast morphological changes of the growing and retracting protrusions (and the displacements of the surrounding matrix) requires a minute to second time scale resolution (see S2 Video). Applying our fast 4D displacement microscopy methodology to our *in vitro* model of sprouting angiogenesis allows monitoring displacement fields not only at sufficiently high temporal resolution (1 minute) but also for multiple sprouts simultaneously (Fig 2 and S3–S5 Videos) captured within a 200 x 200 x 200 μm^3^ volume with voxel sizes of 0.39 x 0.39 x 1 μm^3^. For comparable spatial resolution and image quality, this is 20 times faster than with a conventional confocal microscope (measured here for a Fluoview FV1000, Olympus, Tokyo, Japan). The few other groups investigating displacements around *in vitro* sprouts have reported temporal resolutions of 20 minutes for approximately half of the stack size [36] or 15 seconds for only a single plane [35]. Absolute displacements (**Fig 2A**) unveil that adjacent sprouts invading from a dense layer of cells display qualitatively similar matrix displacement fields. The general pulling activity at the sprout protrusions is oriented towards the sprout base, although with variable magnitude (between 1 μm and 6 μm, see S3 Video) among sprouts. At the same time, the cell layer induces large displacements at the base of the sprouts directed towards the sprout tip. While absolute displacements (t_ref_ = t_fix_) inform us on the overall magnitude and direction of sprout-matrix mechanical interactions, key short-term dynamics are hidden by the combined activity of multiple sprouts. Short-term dynamics of cell-matrix mechanical interactions were unmasked by computing incremental displacements (t_ref_ = t_i_—*τ*) (Methods and S5 Fig) for every timepoint *t*_i_ with a time-lag *τ* of 5 minutes. Fast clustered fluctuations of displacements now vary spatially, temporally and from sprout to sprout (Fig 2B and S6 Video). Quantifying the incremental matrix deformations in close vicinity of each segmented sprout confirmed these observations. At timepoint *t =* 40 min., sprout (viii) is particularly displacing the matrix (3 μm) while others are stalled. (Fig 2F and S8 Fig). All together these results reveal that neighbouring sprouts invade collectively (within 24 hours) but in a non-synchronous, highly dynamic fashion; which is in line with current studies reporting that asynchronous selection of tip cells–regulated by asynchronous individual cell protein dynamics—is required for normal network formation [64–66].

The computational methods in the SPIM-based displacement microscopy were further extended to capture changes of sprout morphological features (Methods) and relate them to matrix displacements here illustrated with selected sprout (ii) from Fig 2C for timepoints t *=* 200 min to t *=* 255 min (Fig 2D, 2E and 2G). Colour-coding the contour of the Z-projected cell mask over time (Fig 2D) allows the inspection of the sprout morphological evolution at a glance. Note that the high temporal resolution provided by SPIM together with the region-based local analysis allows capturing subtle morphological changes such as the time-varying retraction (length and velocity) of a single protrusion upon the addition of CytoD (at t *=* 135 min.) to obtain a stress-free state (at t *=* 255 min.). Matrix displacements around the same protrusion (Fig 2E) gradually decrease alongside with the protrusion retraction (Fig 2G).

**Fig 3:**
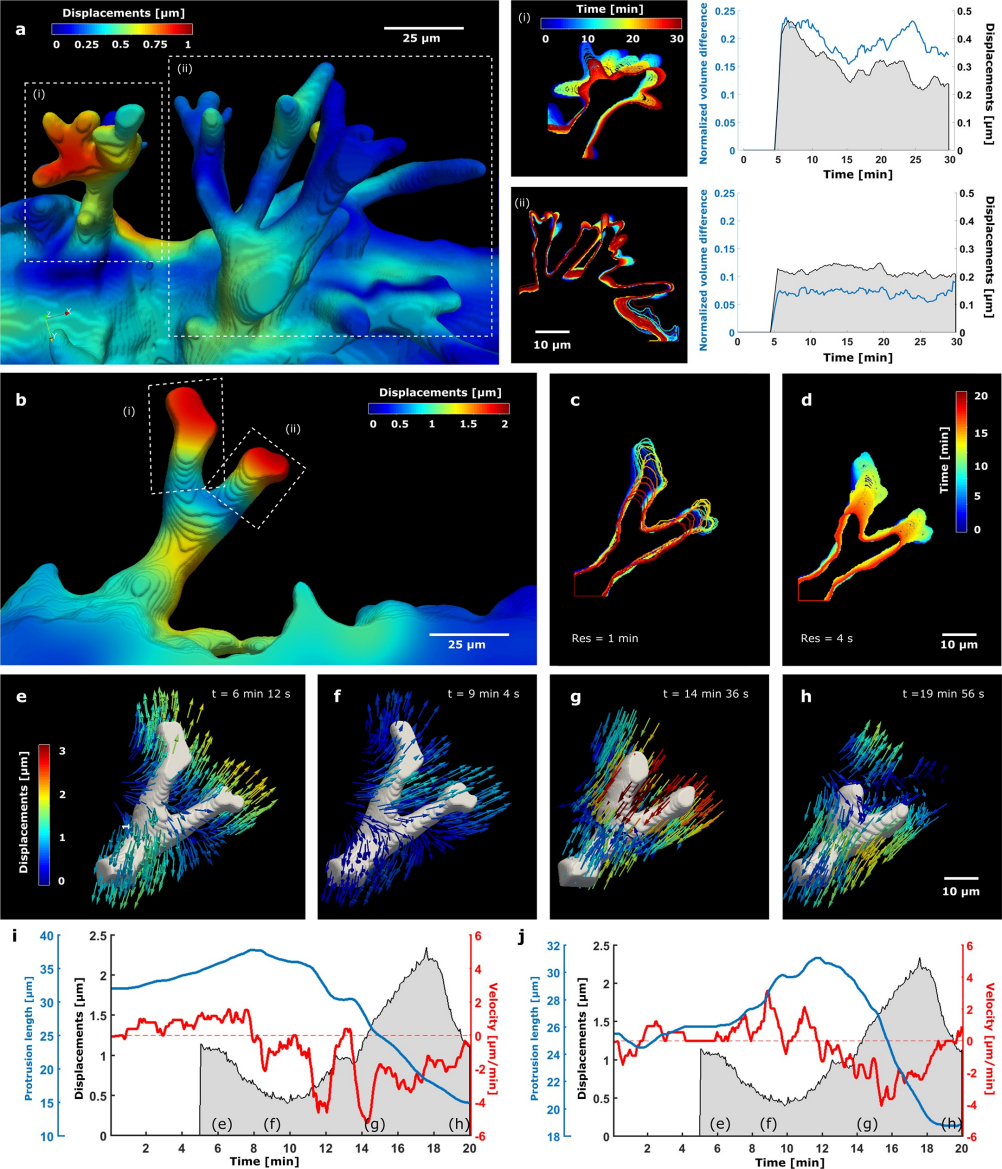
Fast 4D displacement microscopy around dynamic sprout protrusions. **(a)** 3D rendering of sprouts imaged with fast local SPIM scanning (every 10 seconds), with the magnitude of local incremental matrix displacements (t_ref_ = ti− 5 min) mapped on the surface. Contour evolution plot and activity profile of an *active* (i) and *stalled* sprout (ii) (see text for selection criteria) illustrate the positive correlation (S1 Table) between the normalized sprout volume difference and the incremental rms displacements around the selected sprouts. **(b)** 3D rendering of a retracting sprout imaged every 4 seconds with the local displacement magnitudes mapped on the surface. **(c, d)** Contour evolution plots showing shape dynamics for temporal resolutions of 1 minute and 4 seconds, respectively. **(e-h)** Local incremental displacement fields (t_ref_ = ti− 5 min) at indicated timepoints. **(i, j)** Activity profiles of protrusions (i) and (ii) of the sprout in (b). Protrusion (i) shows the highest inverse correlation of its length with the local incremental rms displacements (S1 Table).

While SPIM allows for mapping displacements of multiple adjacent sprouts with subcellular detail—displacements within voxel sizes of 0.39 x 0.39 x 1 μm^3^ were calculated around sprout protrusions (subcellular sized cellular extensions)—the system also provides the flexibility to scan more local, smaller volumes (~200 x 200 x 30 μm^3^) and with improved temporal resolution of every 10 (Fig 3A) or every 4 seconds (Fig 3B–3J). This fast-local scanning is particularly relevant for investigating fast cell morphological dynamics. From a region with adjacent sprouts (Fig 3A), the most *active* (i) and *stalled* sprouts (ii) were identified based on the incremental displacement fields (*τ* = 5 min) (see contour plot in Fig 3A and S6 Video), and defined as the sprouts with highest and lowest local average displacements maximum (in 5 minutes), which was up to 0.44 μm for the active one, and up to 0.25 μm for the stalled one. Note that co-existing active and stalled behaviour exposes the non-synchronicity of neighbouring sprouts described above. Sprout shape dynamics (contour evolution plots, Fig 3A) were quantified by monitoring the normalized volume difference over time (see Methods) and compared to the local incremental displacements, showing moderate to high correlations (see S1 Table) between sprout morphological changes and surrounding matrix deformations. Note that these correlations are measured for a single sprout, with multiple complex-shaped protrusions contributing to the displacement field.

For refined metrics, we hence fast-scan isolated sprouts with more sharply defined protrusions and perform local displacement microscopy analysis (Fig 3B–3H and S7 Video). Contour evolution plots illustrate the image acquisition improvement when switching from scanning with 1 minute resolution used for large volumes (Fig 2D and Fig 3C) to almost continuously capturing a single sprout by locally scanning every 4 seconds (Fig 3D). This is also illustrated in S8 Video. In absence of a CytoD induced stress-free state, we focus on incremental matrix displacements (*τ* = 5 min) to assess protrusion dynamics, while keeping a temporal resolution of 4 seconds (see Methods).

Quantification of the protrusion length and the velocity [67] of its change confirms a time-varying retraction process along with an increase of the local incremental displacements around the protrusion (Fig 3I and 3J). This (inverse) relation between protrusion length and local incremental matrix displacement is particularly strong for protrusion (i) (Fig 3B and 3I and S1 Table). In Fig 3E–3H, the sprout is extending its protrusions while the fluorescent beads move away from the protrusion, followed by the main retraction event (~20 micron between t *=* 8 min and t *=* 20 min, see Fig 3I and 3J) during which the fluorescent beads (S9 Video left) move towards the protrusion. Together with the displacement field (S9 Video right), these observations lead to the conclusion that the observed main retraction event is associated with an increase of contractile forces in the protrusion, which are pulling on the matrix. As such, it is different from retraction events observed after CytoD application (Fig 2D and 2G), which leads to protrusion shortening accompanied by a gradual reduction of both absolute and incremental displacements (see displacement pattern comparison in S9 Fig).

## Conclusion and perspectives

In summary, the high temporal resolution of SPIM-based displacement microscopy allows correlating cell-matrix mechanical interaction to morphological changes at minute to second time scales. Extending displacement microscopy with incremental displacement calculations revealed subtle cell-matrix interactions that would have been overlooked by mere absolute displacement calculations. The possibility to simultaneously follow several large multicellular systems at subcellular level and with high temporal resolution opens new perspectives in mechanobiology, such as for monitoring cell-matrix interactions during angiogenesis [56,67] as well as other multicellular systems such as organoids [68]. Studying other fast subcellular sprouting processes, such as adhesion remodeling, would also be possible at the current imaging parameters, with average adhesion lifetimes reported to be of the order of ~15 min [69]. We believe that even faster processes—such as the protein turnover within apparently stable focal adhesions at the second to millisecond time scale [70,71]—could also be examined with SPIM by reducing the imaged volume. In conclusion, our results demonstrate the rich potential of SPIM-based dynamic displacement imaging for understanding sprout development in angiogenesis.

## Supporting information

S1 Fig SPIM microscope. **(a)** Schematic representation of the excitation unit of the SPIM microscope. L1 and L2 form a beam expander, followed by a galvanometric mirror G for beam deflection. L3 and L4 work as scan and tube lens. At the end of the beam path, an illumination objective (NA = 0.3) excites the sample. **(b)** Detection unit of the SPIM microscope. The sample is imaged by a water dipping objective lens. The dichroic filter F3 allows for two channel detection of red and green emission light, while F1 and F2 block residual excitation light for both channels, T is a tube lens. **(c)** (left) Proportional integral derivative controller to heat up the medium and maintain a temperature of 37° C. This controller was developed on an Arduino Uno microcontroller board. A thermistor electronic element measures the temperature and gives feedback to the Arduino. (right) Temperature stability of the setup, after pre-heating the system.
(TIF)

S2 Fig SPIM setup and microscopy chamber used for the presented experiments. **(a)** Overview of the setup with the two cameras as well as the optical path to generate the light sheet. **(b)** Similar overview as in **a** but from above. **(c)** The sample is held in place by a motorized micrometer stage after the surrounding cultivation medium in the imaging chamber is warmed up.
(TIF)

S3 Fig Schematic of the 3D *in vitro* angiogenesis sample design, compatible with SPIM-based displacement microscopy. **(a)** A fluoroplastic polymer tube is closed at one end with 2-component epoxy glue and sterilized with UV light. **(b)** A collagen hydrogel embedded with a pro-angiogenic factor and fluorescent beads is polymerized inside the container and **(c)** subsequently covered with human umbilical vein endothelial cells (HUVECs) which, after settling and adhering, **(d)** invade the collagen to form in *vitro* angiogenic sprouts (see Methods). Abundant medium is added to the samples after a waiting period of 15 minutes. Grey: tube; purple: sealing; blue: collagen; yellow: fluorescent beads; brown: endothelial cells; pink: growth medium.
(TIF)

S4 Fig Chemically-induced sprout relaxation. **(a)** Schematic of the cytochalasin D diffusion required for chemically inducing the stress-free state of the sprouts **(b)** Diffusion of a fluorescent molecule (DAPI) through the SPIM sample setup in function of time. Representative graph of the fluorescence intensity signal in time. Red curve, data fitted to a sigmoidal curve. **(c)** Boxplot of turning points of the fitted sigmoidal curves in **b**. The average turning point was approximately after 60 minutes. Data collected from 5 independent experiments. **(d)** Relaxation curve. Full field rms displacements from 4 independent experiments, each normalized to its maximum, showing sprout relaxation in function of time. Black arrow, addition of cytochalasin D at t *=* 15 minutes; red asterisk, expected start of the relaxation at t *=* 75 minutes.
(TIF)

S5 Fig Absolute versus incremental displacement calculations for different hypothetical scenarios of cellular pushing and pulling activity. **(a)** Cell-matrix mechanical interactions are monitored by live imaging of cells (in brown) and embedded fluorescent beads (shades of blue, colour-coded for time). Time-dependent bead displacements capture matrix deformations induced by angiogenic sprouts invading from a cellular layer. **(b)** Acquired 4D images are processed to extract the cell mask (in white) and non-rigid image registration is used to compute cell-induced matrix displacement fields over time. For each time point t_i_, absolute displacements (grey arrows) result from the comparison of the bead positions in the stressed matrix (in yellow) with the position of the beads at a reference relaxed end state t_relax_ (in blue) obtained after inducing chemical relaxation of cells. Alternatively, incremental displacements (green arrow) result from the registration of sequential stressed states that are spaced *τ* time points apart; where the bead positions at t_i_ (in yellow) are compared with their corresponding positions at ti−*τ* (in orange). Both approaches are complementary: while absolute displacements provide quantitative information on the magnitude and direction of cell-induced displacements over time, the magnitude of the incremental displacements better reveals local changes in the displacement field within the selected time-lag *τ*. **(c-d)** The direction of the incremental displacements only cannot discriminate between pushing or pulling activity of cells; instead, it should be considered together with the absolute displacements or, alternatively, with the cell activity over time. Absolute displacements induced by pulling and pushing cells are directed inwards **(c.i)** and outwards **(d.i)**, respectively. When the direction of incremental displacements matches the direction of absolute displacements, the cell reinforces either its pulling **(c.ii)** or pushing **(d.ii)** activity. In contrast, if the incremental and absolute displacements present opposite directions, the cell decreases either its pulling **(c.iii)** or pushing **(d.iii)** activity. It must be remarked that the absolute displacement fields around sprout tips as observed by SPIM-based displacement microscopy are indicative of pulling activity (as depicted in **c**).
(TIF)

S6 Fig STICS analysis consecutively in three dimensions confirms sample and system stability, a prerequisite to recover reliable displacements in cellular samples. **(a)** STICS correlation map in an (xy)- plane of a representative sample for a temporal lag of 4 seconds in a 3D representation. **(b)** Correlation maps of (xy)-, (zy)- and (zx)- crossections. **(c, d)** Correlation map features (of two more representative samples) in function of time-lags show fluorescent bead stability for a wide range of time-lags: every 0, 3, 6, 12 and 24 minutes **(c)** and every 0, 15, 30, 60 ad 120 seconds **(d)**. These features include the peak value of the correlation map (corrPeak) and the parameters of a fitted 3D Gaussian function: amplitude (gaussPeak), mean (muX, muY and muZ) and standard deviation (sigmaX, sigmaY and sigmaZ).
(TIF)

S7 Fig Kymographic representation of the 3D STICS analysis as a visual control for fluorescent bead attachment to the collagen. **(a)** First, a section is taken from the volume and aligned over time. **(b)** These sections are cross-sectioned resulting again in a stack with x- or y- and t-axis. **(c)** Examples of the different possible results from the STICS analysis. If no bead motion occurred, the beads are stable over time, resulting in straight lines. (**d**) Two general results are possible when the beads do move. The left image is a result of random bead diffusion and the right image displays a shift from the beads in the gel which could not be corrected by drift correction. **(e-g)** Kymographic representation (in x-direction) of acellular control experiments with a collagen concentration of 0.5 mg/ml **(d)**, 1 mg/ml **(e)** and 1.5 mg/ml **(f)**. Kymographs per condition are representative for results from three independent collagen batches.
(TIF)

S8 Fig Local data processing around individual sprouts. SPIM-based displacement microscopy facilitates analysis of large data volumes at subcellular level. (top) The segmented sprouts from Fig 2C, indicated with Roman numbers. (bottom) Matrix displacements in time given by the incremental rms displacements (*τ* = 5 min) in closest vicinity of each sprout between timepoints t *=* 30 min and t *=* 145 min. Segmented sprouts rendered with MatLab. Acquired volume of approximately 200 x 200 x 200 μm^3^ every minute (*Res* = 1 min). Drift- compensated data volume of 190 x 190 x 170 μm^3^.
(TIF)

S9 Fig Comparison of protrusion retractions: sprout pulling versus sprout relaxing. **(a)** (top) A sketch showing a retracting sprout and (i) its surrounding beads moving closer to the sprout in time. Typically, an actively pulling sprout results in (ii) absolute and (iii) incremental displacement fields with arrows in the same direction as the retraction, indicative of pulling activity that is increasing with time. (bottom) Temporal projection of microscopy images of a representative pulling sprout (see text Fig 3B) retracting its protrusion ~20 micron between t *=* 8 min and t *=* 20 min), with its surrounding beads and a corresponding incremental (τ = 5 min) displacement field at t *=* 15 min. **(b)** (top) A sketch showing a relaxing sprout and (i) its surrounding beads moving away from the sprout in time. Typically, a relaxing sprout results in (iii) incremental displacement fields with arrows in the opposite direction as the retraction, while (ii) absolute displacements still indicate pulling activity (that is decreasing with time). (bottom) Temporal projection of microscopy images of a representative relaxing sprout (data crop from sprout in Fig 2D of main text), retracting its protrusion ~30 micron between t *=* 206 min and t *=* 232 min, with its surrounding beads and a corresponding incremental (τ = 5 min) displacement field at t *=* 225 min.
(TIF)

S1 Table Correlations between displacements and morphology. Correlations between incremental matrix displacements and sprout morphological changes. Auto-correlation values indicate correlations between the local rms displacements and morphological changes (normalized volume difference or protrusion length) of the same sprout, while cross-correlation values indicate correlations between rms displacements from the displayed sprout and morphological changes from another (not displayed) sprout grown in similar conditions. Correlations are indicated in italic.
(DOCX)

S1 Video Timelapse of the chemically-induced sprout relaxation. An *in vitro* angiogenic sprout imaged with Selective Plane Illumination Microscopy (SPIM) for approximately four hours. Sprout relaxation is induced after 15 minutes (presence of the relaxing agent in the system indicated with an asterisk), to remove deformations from the collagen hydrogel, and obtain a stress-free hydrogel state as a reference state for calculating absolute displacements.
(AVI)

S2 Video Increasing the temporal resolution from 5 to 1 minute for imaging fast morphological sprout dynamics. A sprout imaged with the SPIM setup shown for a 5 minute interval (left) and a 1 minute interval (right) as temporal resolution. Acquired volume of approximately 200 x 200 x 200 μm^3^. Drift—compensated data volume of 166 x 187 x 183 μm^3^.”
(AVI)

S3 Video Timelapse of the full field absolute displacements around *in vitro* angiogenic sprouts. The segmented sprouts and downsampled displacement vectors are rendered with Paraview. The size and color of the arrows reflect the displacement magnitude. The full data range of displacement magnitudes is shown, except for a lower bound at 0.2 micron for improved pattern visualization. Acquired volume of approximately 200 x 200 x 200 μm^3^. Drift- compensated data volume of 190 x 190 x 170 μm^3^. The temporal resolution *Res* is 1 minute.
(AVI)

S4 Video Timelapse of the local absolute displacement field around *in vitro* angiogenic sprouts. Local displacements up to 15 pixels (~5 μm) around the segmented sprouts are taken into account. The displacement data, represented by the arrows, is downsampled for visibility. The color of the arrows reflects the magnitude of the displacement vector, while the size of the arrows is kept uniformly, to highlight the gradual distributions of the displacement magnitudes. The full data range of sprout displacement magnitudes is shown, except for a lower bound at 0.2 μm displacements for improved pattern visualization. Acquired volume of approximately 200 x 200 x 200 μm^3^. Drift- compensated data volume of 190 x 190 x 170 μm^3^. The temporal resolution Res is 1 minute.
(AVI)

S5 Video Timelapse of the magnitude of the absolute displacements closest to the sprout mapped on the segmented sprout surface. The full range of sprout displacement magnitudes is shown, and discretized for pattern recognition. Acquired volume of approximately 200 x 200 x 200 μm^3^. Drift- compensated data volume of 190 x 190 x 170 μm^3^.
(AVI)

S6 Video **Timelapse of the absolute (left) and incremental (right) full field displacements around *in vitro* angiogenic sprouts.** While absolute displacements provide quantitative information on the magnitude, direction and global distribution of cell-induced displacements over time, the magnitude of the incremental displacements reveals the instantaneous spatial distribution of local changes in the displacement field within the selected time-lag *τ* = 5 minutes, while keeping a temporal resolution of 1 minute. Acquired volume of approximately 200 x 200 x 200 μm^3^. Drift- compensated data volume of 190 x 190 x 170 μm^3^. The temporal resolution *Res* is 1 minute.
(AVI)

S7 Video **Timelapse of *stalled* (left) and *active* (right) sprout protrusions (see Fig 3A of main text), displaying different activity profiles in terms of morphological changes and incremental displacement fields.** The data was collected for a volume of approximately 200 x 200 x 30 micron^3^ with a temporal resolution *Res* of 10 seconds, for a total of 30 minutes. The incremental displacements were calculated with a time-lag *τ* = 5 min. Data crops were made for optimized fast local displacement microscopy. Drift—corrected data volume of the left crop = 82 x 95 x 46 μm^3^. Drift- compensated data volume of the right crop = 46 x 60 x 46 μm^3^.
(AVI)

S8 Video Increased temporal resolution from 1 minute to 4 seconds for imaging fast morphological protrusion dynamics. A protrusion imaged with the SPIM setup shown for a 1 minute interval (left) and a 4 seconds interval (right) as temporal resolution. Drift—compensated data volume of the crop = 61 x 73 x 18 μm^3^.
(AVI)

S9 Video Timelapse of retracting sprout protrusions. (left) Drift—compensated timelapse, with the cell and fluorescent bead channels overlaid, showing the sprout interacting with the matrix. The data volume was collected with a temporal resolution of 4 seconds, for a total of 20 minutes. (right) Local incremental displacement field (*τ* **=** 5 min) around the sprout protrusions, represented by uniformly distributed arrows color-coded for the magnitude of the displacement vector. The upper bound of the displacement range was chosen at 2 μm and kept constant during the timelapse for consistency (all displacements > 2 μm are shown in red). Drift- compensated data volume of the crop **=** 61 x 73 x 18 μm^3^.
(AVI)
