# Supplementary figures and images for: Fast quantitative time lapse displacement imaging of endothelial cell invasion
Source: PLoS One. 2020 Jan 7;15(1):e0227286. doi: 10.1371/journal.pone.0227286 (PMC6946139; doi:10.1371/journal.pone.0227286)

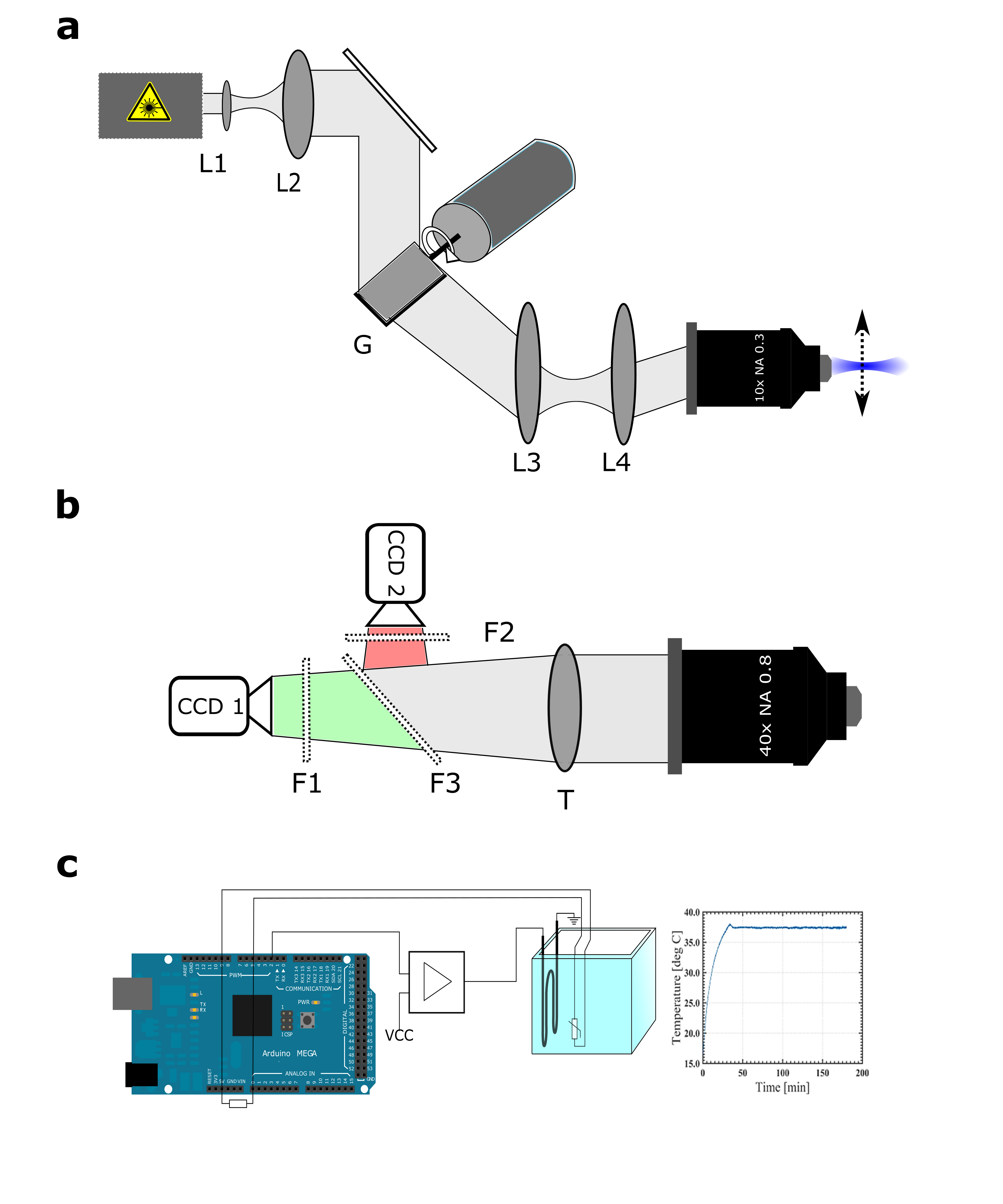

Supplement: S1 Fig — (a) Schematic representation of the excitation unit of the SPIM microscope. L1 and L2 form a beam expander, followed by a galvanometric mirror G for beam deflection. L3 and L4 work as scan and tube lens. At the end of the beam path, an illumination objective (NA = 0.3) excites the sample. (b) Detection unit of the SPIM microscope. The sample is imaged by a water dipping objective lens. The dichroic filter F3 allows for two channel detection of red and green emission light, while F1 and F2 block residual excitation light for both channels, T is a tube lens. (c) (left) Proportional integral derivative controller to heat up the medium and maintain a temperature of 37° C. This controller was developed on an Arduino Uno microcontroller board. A thermistor electronic element measures the temperature and gives feedback to the Arduino. (right) Temperature stability of the setup, after pre-heating the system. (TIF) [file pone.0227286.s001.tif]

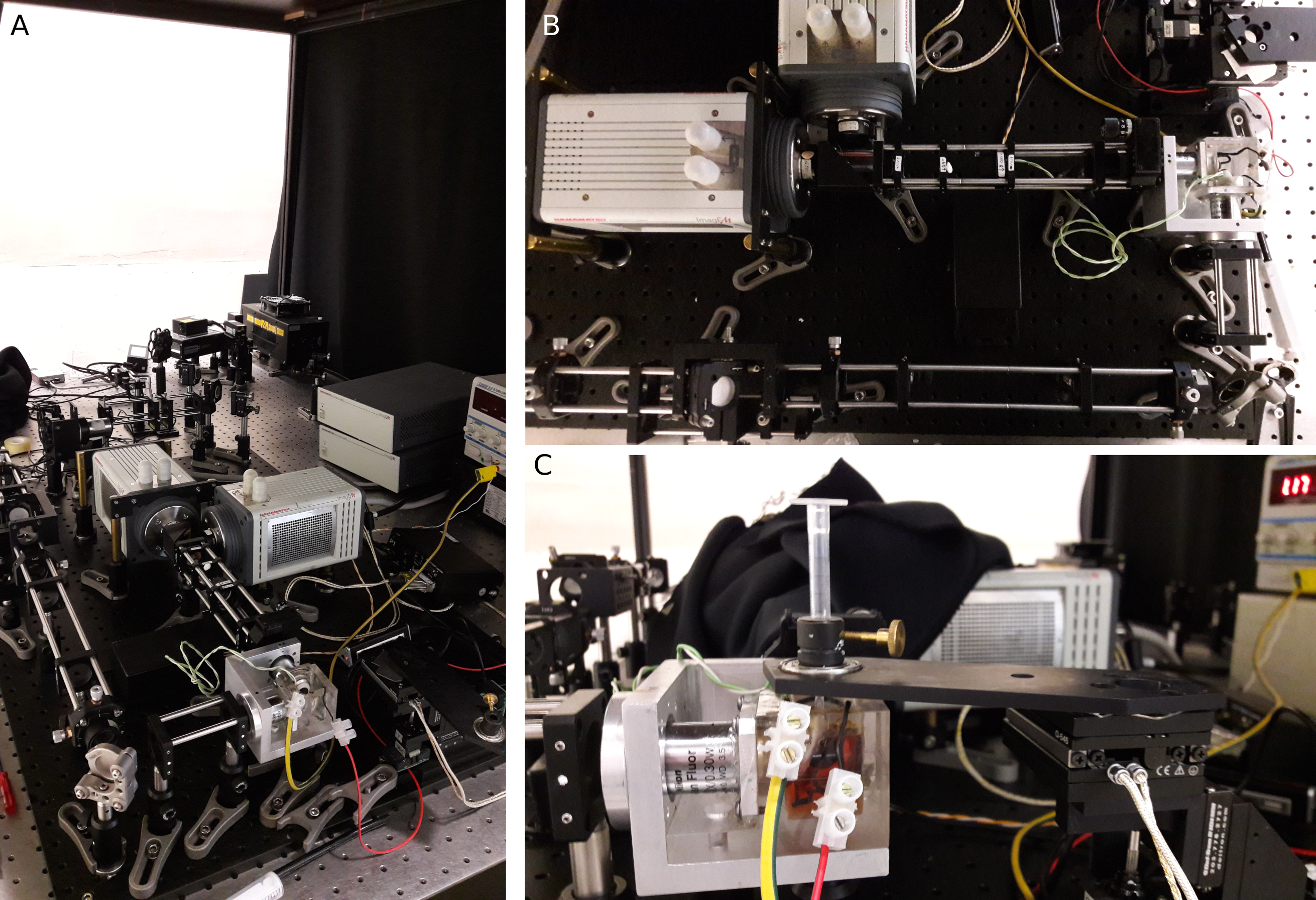

Supplement: S2 Fig — (a) Overview of the setup with the two cameras as well as the optical path to generate the light sheet. (b) Similar overview as in a but from above. (c) The sample is held in place by a motorized micrometer stage after the surrounding cultivation medium in the imaging chamber is warmed up. (TIF) [file pone.0227286.s002.tif]

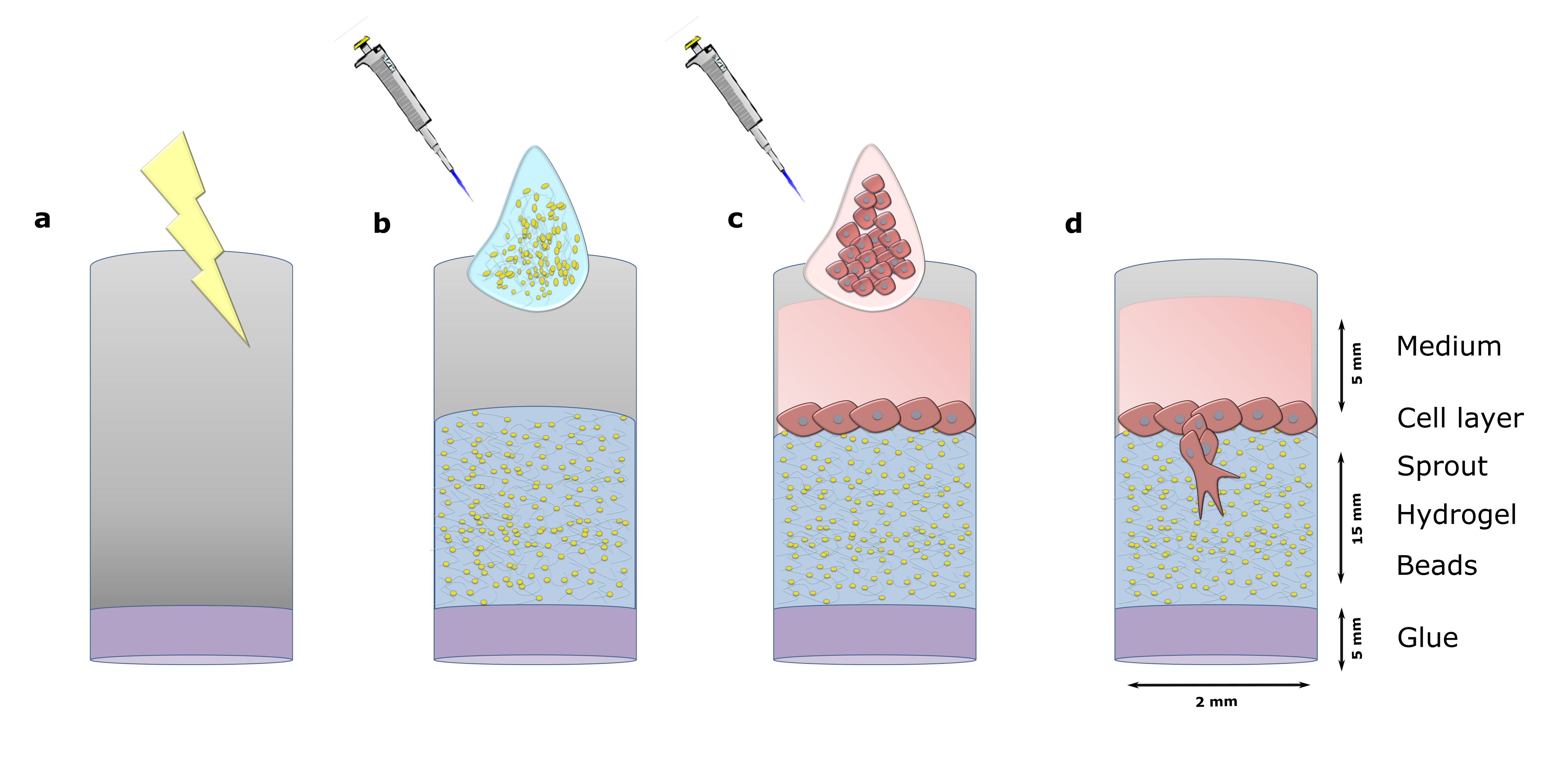

Supplement: S3 Fig — (a) A fluoroplastic polymer tube is closed at one end with 2-component epoxy glue and sterilized with UV light. (b) A collagen hydrogel embedded with a pro-angiogenic factor and fluorescent beads is polymerized inside the container and (c) subsequently covered with human umbilical vein endothelial cells (HUVECs) which, after settling and adhering, (d) invade the collagen to form in vitro angiogenic sprouts (see Methods). Abundant medium is added to the samples after a waiting period of 15 minutes. Grey: tube; purple: sealing; blue: collagen; yellow: fluorescent beads; brown: endothelial cells; pink: growth medium. (TIF) [file pone.0227286.s003.tif]

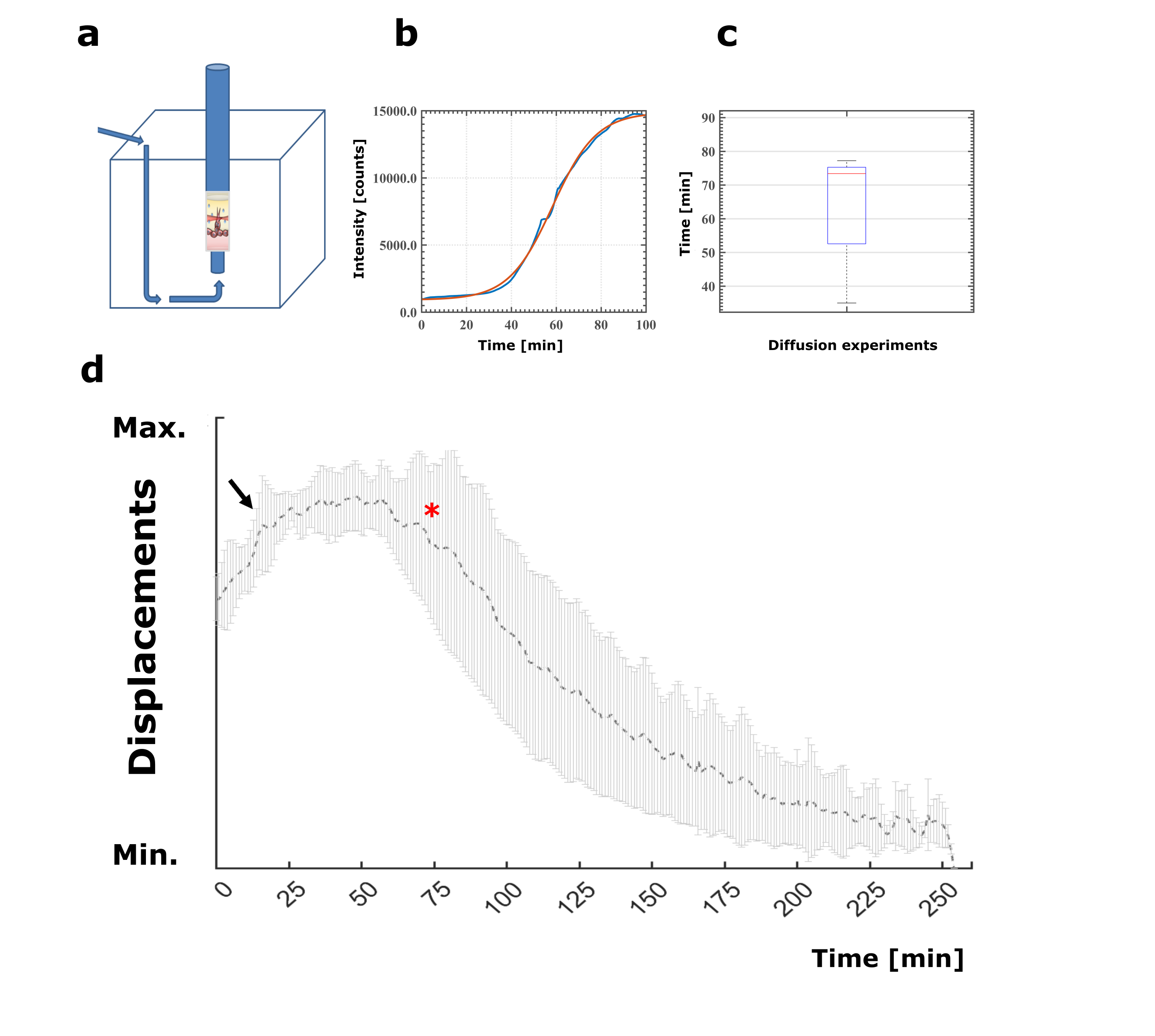

Supplement: S4 Fig — (a) Schematic of the cytochalasin D diffusion required for chemically inducing the stress-free state of the sprouts (b) Diffusion of a fluorescent molecule (DAPI) through the SPIM sample setup in function of time. Representative graph of the fluorescence intensity signal in time. Red curve, data fitted to a sigmoidal curve. (c) Boxplot of turning points of the fitted sigmoidal curves in b. The average turning point was approximately after 60 minutes. Data collected from 5 independent experiments. (d) Relaxation curve. Full field rms displacements from 4 independent experiments, each normalized to its maximum, showing sprout relaxation in function of time. Black arrow, addition of cytochalasin D at t = 15 minutes; red asterisk, expected start of the relaxation at t = 75 minutes. (TIF) [file pone.0227286.s004.tif]

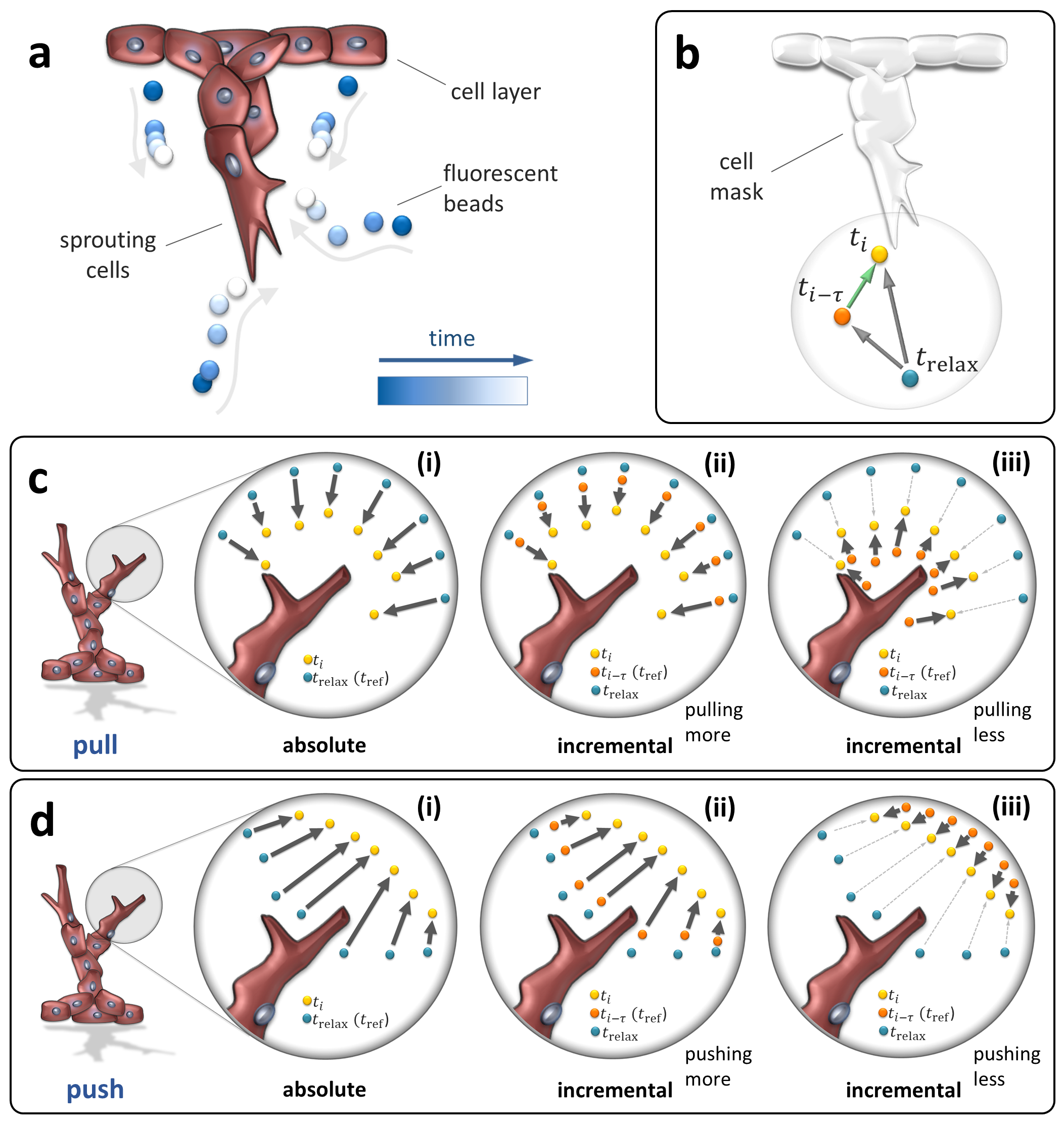

Supplement: S5 Fig — (a) Cell-matrix mechanical interactions are monitored by live imaging of cells (in brown) and embedded fluorescent beads (shades of blue, colour-coded for time). Time-dependent bead displacements capture matrix deformations induced by angiogenic sprouts invading from a cellular layer. (b) Acquired 4D images are processed to extract the cell mask (in white) and non-rigid image registration is used to compute cell-induced matrix displacement fields over time. For each time point ti, absolute displacements (grey arrows) result from the comparison of the bead positions in the stressed matrix (in yellow) with the position of the beads at a reference relaxed end state trelax (in blue) obtained after inducing chemical relaxation of cells. Alternatively, incremental displacements (green arrow) result from the registration of sequential stressed states that are spaced τ time points apart; where the bead positions at ti (in yellow) are compared with their corresponding positions at ti−τ (in orange). Both approaches are complementary: while absolute displacements provide quantitative information on the magnitude and direction of cell-induced displacements over time, the magnitude of the incremental displacements better reveals local changes in the displacement field within the selected time-lag τ. (c-d) The direction of the incremental displacements only cannot discriminate between pushing or pulling activity of cells; instead, it should be considered together with the absolute displacements or, alternatively, with the cell activity over time. Absolute displacements induced by pulling and pushing cells are directed inwards (c.i) and outwards (d.i), respectively. When the direction of incremental displacements matches the direction of absolute displacements, the cell reinforces either its pulling (c.ii) or pushing (d.ii) activity. In contrast, if the incremental and absolute displacements present opposite directions, the cell decreases either its pulling (c.iii) or pushing (d.i [file pone.0227286.s005.tif]

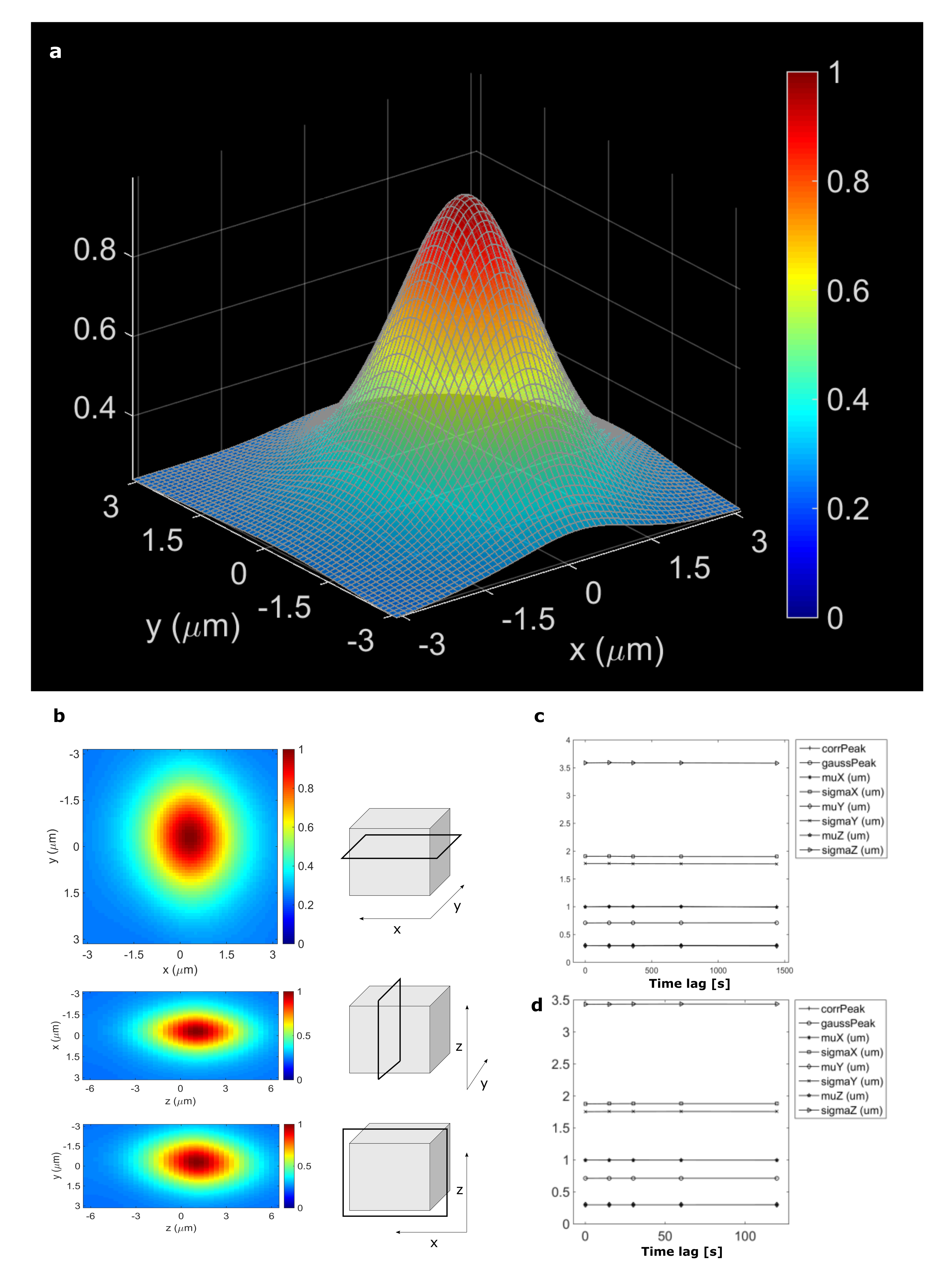

Supplement: S6 Fig — (a) STICS correlation map in an (xy)- plane of a representative sample for a temporal lag of 4 seconds in a 3D representation. (b) Correlation maps of (xy)-, (zy)- and (zx)- crossections. (c, d) Correlation map features (of two more representative samples) in function of time-lags show fluorescent bead stability for a wide range of time-lags: every 0, 3, 6, 12 and 24 minutes (c) and every 0, 15, 30, 60 ad 120 seconds (d). These features include the peak value of the correlation map (corrPeak) and the parameters of a fitted 3D Gaussian function: amplitude (gaussPeak), mean (muX, muY and muZ) and standard deviation (sigmaX, sigmaY and sigmaZ). (TIF) [file pone.0227286.s006.tif]

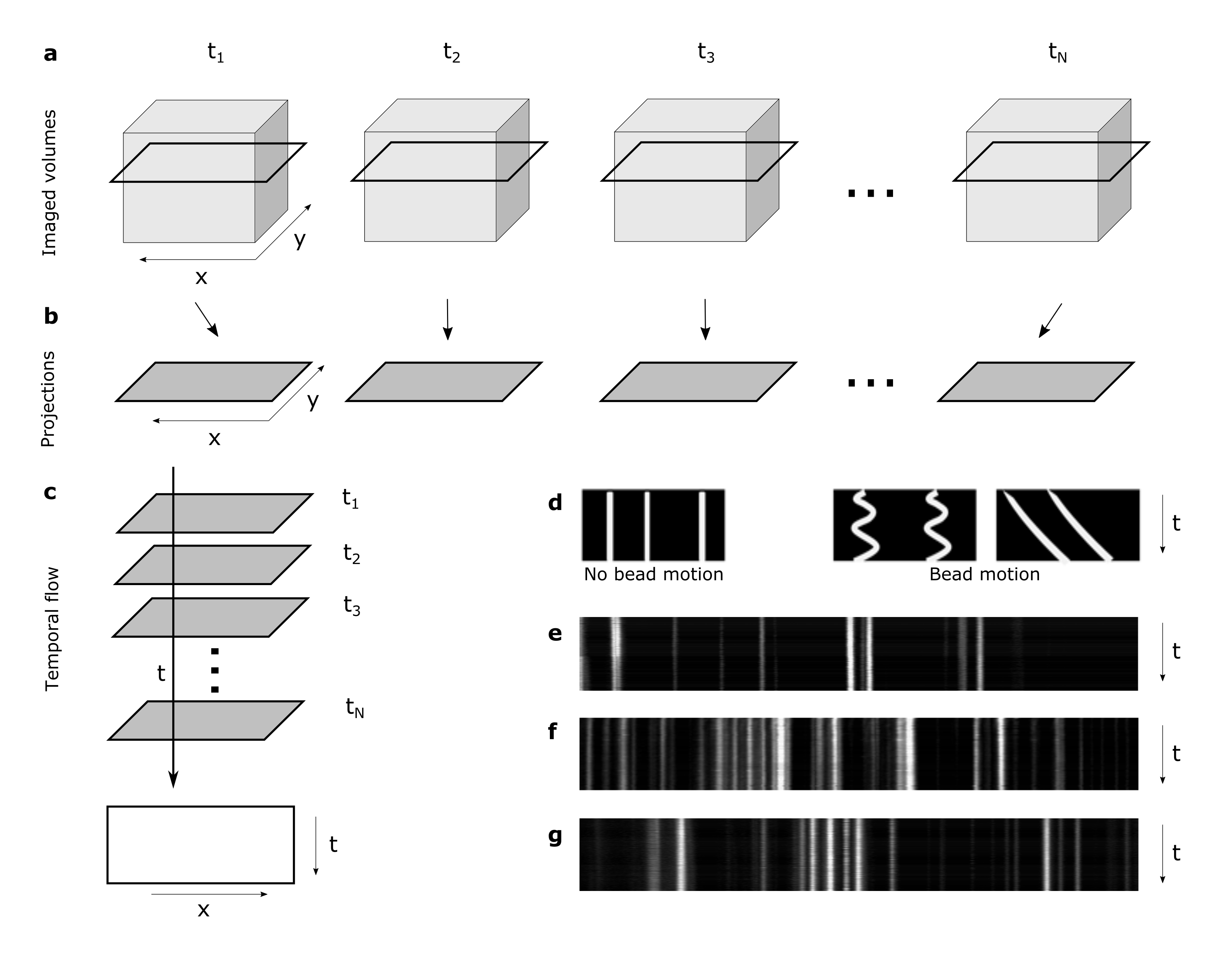

Supplement: S7 Fig — (a) First, a section is taken from the volume and aligned over time. (b) These sections are cross-sectioned resulting again in a stack with x- or y- and t-axis. (c) Examples of the different possible results from the STICS analysis. If no bead motion occurred, the beads are stable over time, resulting in straight lines. (d) Two general results are possible when the beads do move. The left image is a result of random bead diffusion and the right image displays a shift from the beads in the gel which could not be corrected by drift correction. (e-g) Kymographic representation (in x-direction) of acellular control experiments with a collagen concentration of 0.5 mg/ml (d), 1 mg/ml (e) and 1.5 mg/ml (f). Kymographs per condition are representative for results from three independent collagen batches. (TIF) [file pone.0227286.s007.tif]

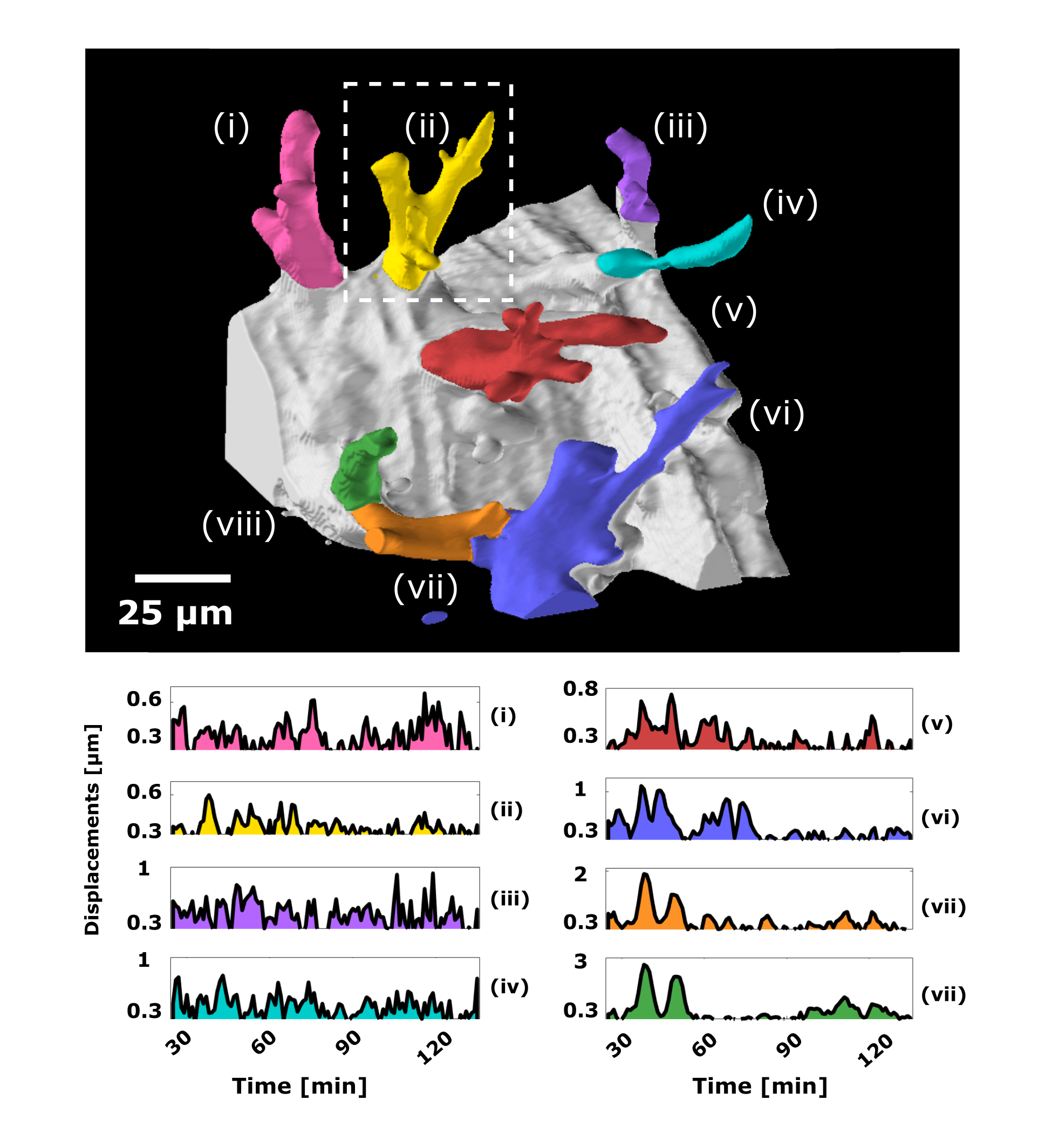

Supplement: S8 Fig — SPIM-based displacement microscopy facilitates analysis of large data volumes at subcellular level. (top) The segmented sprouts from Fig 2C, indicated with Roman numbers. (bottom) Matrix displacements in time given by the incremental rms displacements (τ = 5 min) in closest vicinity of each sprout between timepoints t = 30 min and t = 145 min. Segmented sprouts rendered with MatLab. Acquired volume of approximately 200 x 200 x 200 μm3 every minute (Res = 1 min). Drift- compensated data volume of 190 x 190 x 170 μm3. (TIF) [file pone.0227286.s008.tif]

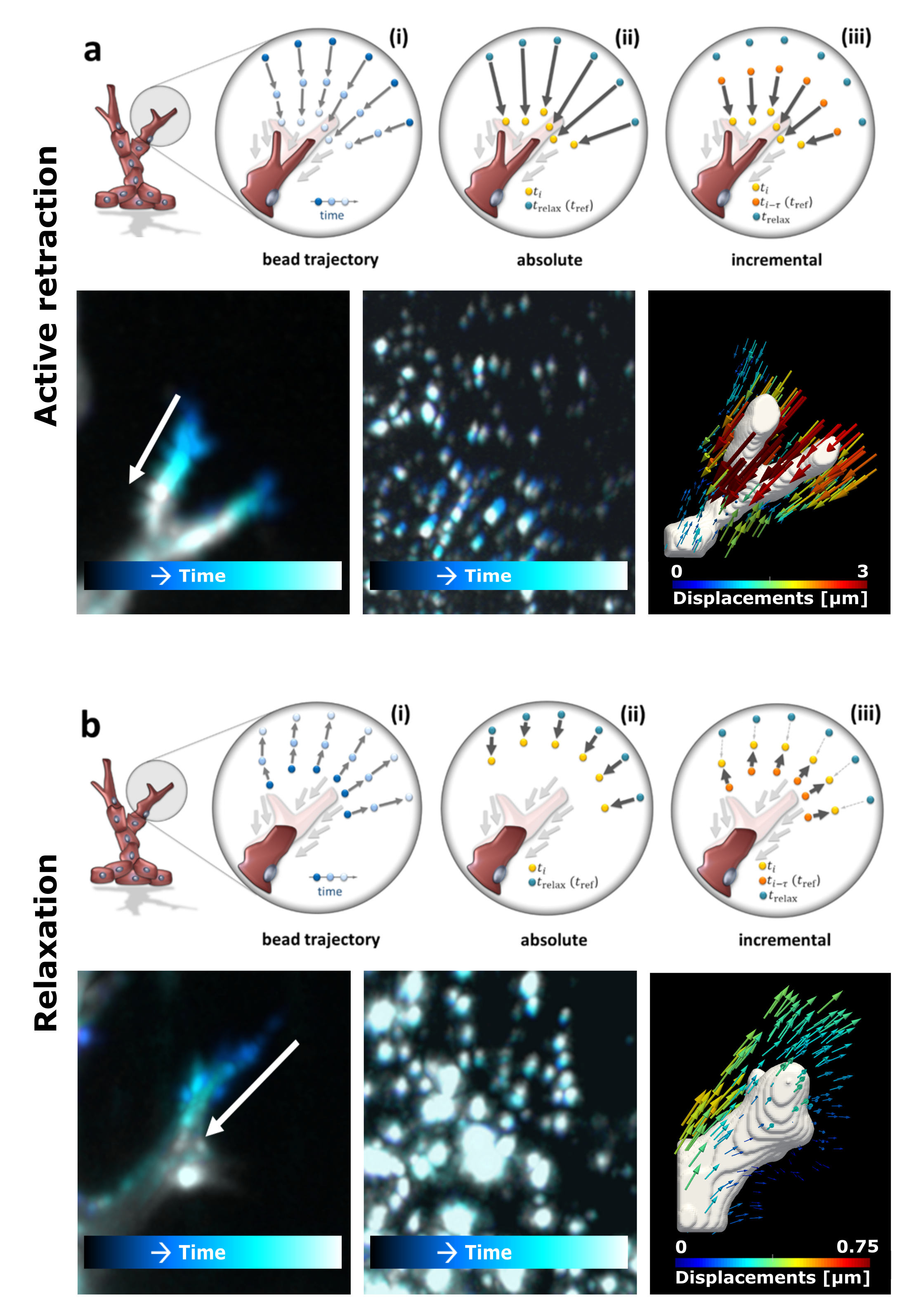

Supplement: S9 Fig — (a) (top) A sketch showing a retracting sprout and (i) its surrounding beads moving closer to the sprout in time. Typically, an actively pulling sprout results in (ii) absolute and (iii) incremental displacement fields with arrows in the same direction as the retraction, indicative of pulling activity that is increasing with time. (bottom) Temporal projection of microscopy images of a representative pulling sprout (see text Fig 3B) retracting its protrusion ~20 micron between t = 8 min and t = 20 min), with its surrounding beads and a corresponding incremental (τ = 5 min) displacement field at t = 15 min. (b) (top) A sketch showing a relaxing sprout and (i) its surrounding beads moving away from the sprout in time. Typically, a relaxing sprout results in (iii) incremental displacement fields with arrows in the opposite direction as the retraction, while (ii) absolute displacements still indicate pulling activity (that is decreasing with time). (bottom) Temporal projection of microscopy images of a representative relaxing sprout (data crop from sprout in Fig 2D of main text), retracting its protrusion ~30 micron between t = 206 min and t = 232 min, with its surrounding beads and a corresponding incremental (τ = 5 min) displacement field at t = 225 min. (TIF) [file pone.0227286.s009.tif]
