# Supplementary material for: Fast quantitative time lapse displacement imaging of endothelial cell invasion
Source: PLoS One. 2020 Jan 7;15(1):e0227286. doi: 10.1371/journal.pone.0227286 (PMC6946139; doi:10.1371/journal.pone.0227286)
Supplement: S1 Table — Correlations between incremental matrix displacements and sprout morphological changes. Auto-correlation values indicate correlations between the local rms displacements and morphological changes (normalized volume difference or protrusion length) of the same sprout, while cross-correlation values indicate correlations between rms displacements from the displayed sprout and morphological changes from another (not displayed) sprout grown in similar conditions. Correlations are indicated in italic. (DOCX) [file pone.0227286.s010.docx]

| **SAMPLE & METHOD** | | **COMPARED FEATURES** | | | | **CORRELATIONS** | | |  |  |  |  |  |  |  |
| --- | --- | --- | --- | --- | --- | --- | --- | --- | --- | --- | --- | --- | --- | --- | --- |
| **Fig 3a** | | | **Morphology:**$\boldsymbol{V}_{\text{norm\_dif}}$ | | **Local rms disp** |  | |  | | | | | |  |  |
| Auto-correlation | | | Sprout (i) | | Sprout (i) | *0.6671* | |  | | | |  |  |  |  |
| Auto-correlation | | | Sprout (ii) | | Sprout (ii) | *0.7683* | |  | | | |  |  |  |  |
| Cross-correlation | | | Sprout (i) | | Sprout (ii) | *0.4110* | |  | | | |  |  |  |  |
| Cross-correlation | | | Sprout (ii) | | Sprout (i) | *0.3998* | |  | | | |  |  |  |  |
|  | | |  | |  |  | |  | |  | | | | |  |
| **Fig. 3b** | | | **Morphology:** $\boldsymbol{V}_{\text{norm\_dif}}$ | | **Local rms disp** |  | |  | | |  | |  |  |  |
| Auto-correlation | | | Displayed sprout | | Displayed sprout | *0.8533* | |  | | |  | |  |  |  |
| Auto-correlation | | | Non-displayed sprout | | Non-displayed sprout | *0.8317* | |  | | |  | |  |  |  |
| Cross-correlation | | | Displayed sprout | | Non-displayed sprout | *0.3423* | |  | | |  | |  |  |  |
| Cross-correlation | | | Non-displayed sprout | | Displayed sprout | *0.3338* | |  | | |  | |  |  |  |
|  | | |  | |  |  | |  | |  | | | | |  |
| **Fig. 3b** | | | **Morphology: Length** | | **Local rms disp** |  | |  | |  | | | | |  |
| Auto-correlation | | | Protrusion (i) | | Protrusion (i) | *-0.8363* | |  | |  | | | | |  |
| Auto-correlation | | | Protrusion (ii) | | Protrusion (ii) | *-0.7486* | |  | |  | | | | | |
|  |  | | |  | |  |  | | | | | | | | |
